# Exercise training modalities in patients with type 2 diabetes mellitus: a systematic review and network meta-analysis

**DOI:** 10.1186/s12966-018-0703-3

**Published:** 2018-07-25

**Authors:** Bei Pan, Long Ge, Yang-qin Xun, Ya-jing Chen, Cai-yun Gao, Xue Han, Li-qian Zuo, Hou-qian Shan, Ke-hu Yang, Guo-wu Ding, Jin-hui Tian

**Affiliations:** 1 Department of Social Medicine and Health Management, School of Public Health, Lanzhou University, Lanzhou, 730000 China; 2 The First Clinical Medical College, Lanzhou University, Lanzhou, 730000 China; 3 Evidence-Based Medicine Center, School of Basic Medical Sciences, Lanzhou University, No. 199, Dong gang West Road, Chengguan District, Lanzhou City, Gansu province China

**Keywords:** Type 2 diabetes mellitus, Exercise training, Glycemic control, Cardiovascular risk factors, Weight loss, Frequentist network meta-analysis

## Abstract

**Introduction:**

Current international guidelines recommend aerobic, resistance, and combined exercises for the management of type 2 diabetes mellitus (T2DM). In our study, we conducted a network meta-analysis to assess the comparative impact of different exercise training modalities on glycemic control, cardiovascular risk factors, and weight loss in patients with T2DM.

**Methods:**

We searched five electronic databases to identify randomized controlled trials (RCTs) that compared the differences between different exercise training modalities for patients with T2DM. The risk of bias in the included RCTs was evaluated according to the Cochrane tool. Network meta-analysis was performed to calculate mean difference the ratio of the mean and absolute risk differences. Data were analyzed using R-3.4.0.

**Results:**

A total of 37 studies with 2208 patients with T2DM were included in our study. Both supervised aerobic and supervised resistance exercises showed a significant reduction in HbA1c compared to no exercise (0.30% lower, 0.30% lower, respectively), however, there was a less reduction when compared to combined exercise (0.17% higher, 0.23% higher). Supervised aerobic also presented more significant improvement than no exercise in fasting plasma glucose (9.38 mg/dl lower), total cholesterol (20.24 mg/dl lower), triacylglycerol (19.34 mg/dl lower), and low-density lipoprotein cholesterol (11.88 mg/dl lower). Supervised resistance showed more benefit than no exercise in improving systolic blood pressure (3.90 mmHg lower]) and total cholesterol (22.08 mg/dl lower]. In addition, supervised aerobic exercise was more powerful in improving HbA1c and weight loss than unsupervised aerobic (HbA1c: 0.60% lower; weight loss: 5.02 kg lower) and unsupervised resistance (HbA1c: 0.53% lower) exercises.

**Conclusion:**

Compared with either supervised aerobic or supervised resistance exercise alone, combined exercise showed more pronounced improvement in HbA1c levels; however, there was a less marked improvement in some cardiovascular risk factors. In terms of weight loss, there were no significant differences among the combined, supervised aerobic, and supervised resistance exercises.

**Trial registration:**

Our study protocol was registered with the International Prospective Register of Systematic Reviews (PROSPERO); registration number: CRD42017067518.

**Electronic supplementary material:**

The online version of this article (10.1186/s12966-018-0703-3) contains supplementary material, which is available to authorized users.

## Background

Diabetes affects approximately 382 million adults worldwide, and is predicted to increase to 439 million adults by 2030 [1, 2]. Type 2 diabetes mellitus (T2DM) accounts for 85–95% of all diabetes cases in the world [2]. A sedentary lifestyle is considered as one of the major risk factors for T2DM and its complications [3]. Maintaining an appropriate level of physical activity is an effective strategy for T2DM management [4, 5].

Most patients with T2DM display dyslipidemia, hypertension and hyperinsulinemia, which are associated with metabolic syndrome and will lead to an increased risk of premature cardiovascular disease [6]. Comorbid conditions and complications are considered to determine the quality of life of patients with T2DM [7, 8]. Improved muscular and cardiorespiratory fitness are associated with reduced mortality rates [9–11]. Studies have shown that resistance training exercise can increase muscular strength and improve the control of blood glucose and HbA1c levels [12]. Aerobic exercise can also increase cardiorespiratory fitness and improve the control of blood glucose and HbA1c levels in patients with T2DM [13, 14].

Physical activity has been recommended as an important non-pharmacological therapeutic strategy for the management of T2DM by some major international organizations in this field [15]. Current national and international guidelines recommend aerobic and resistance exercise training for T2DM patients [16–19]. A combination of aerobic exercise and resistance exercise (combined exercise) has been recommended by the European Society of Cardiology [16], American College of Sports Medicine [17], Belgian Physical Therapy Association [18], and Exercise and Sports Science Australia [19]. Thus, multiple exercise training modalities have been recommended by different international organizations. The latest Canadian guideline [20] recommends supervised exercise as an effective modality for improving glycemic control, and weight loss. Different training modalities such as aerobic exercise, resistance exercise, combined exercise, and flexibility training are recommended. The recommendations are thus inconclusive and some bodies specifies supervised exercise in their recommendations.

Several randomized controlled trials (RCTs) and systematic reviews have been conducted to investigate the impact of aerobic or resistance exercise on glycemic control, cardiovascular risk factors and muscle strength in T2DM patients [21–24]. However, it is difficult to determine the superiority of different physical activities using RCTs or pairwise meta-analysis. Network meta-analysis has become increasingly popular to evaluate healthcare interventions, since it allows for estimation of the relative effectiveness among all interventions and rank ordering of the interventions even if head-to-head comparisons are lacking [25].

In the current study, we aimed to compare different exercise training modalities in the improvement of glycemic control, weight loss, and cardiovascular risk factors for patients with T2DM using Frequentist network meta-analysis.

## Methods and analysis

### Registration

Our study protocol was registered with the International Prospective Register of Systematic Reviews (PROSPERO); registration number: CRD42017067518.

### Search strategy

Searches of the PubMed, EMBASE, and Cochrane Central Register of Controlled Trials (CENTRAL) databases were conducted in April 2017. The references of included articles and relevant systematic reviews and meta-analyses were tracked for additional studies. There were no restrictions in terms of the year of publication or publication status. Search terms included: random*, type 2 diabetes, exercise, aerobic exercise, resistance exercise and combined exercise. The search strategy is shown in Additional file 1: Appendix 1.

### Inclusion criteria

Type of participants: We included studies enrolling participants with T2DM aged ≥18 years. Studies including patients with other chronic diseases, children, adolescents or pregnant women were excluded [26].

Type of design: Randomized controlled trials (RCTs).

Type of interventions: We focused on the following eight exercise training modalities: supervised aerobic exercise, unsupervised aerobic exercise, anaerobic exercise, supervised resistance exercise, unsupervised resistance exercise, combined exercise, flexibility exercise, and no exercise. The definition of each intervention is shown in Additional file 1: Appendix 2.

Type of outcomes: Outcomes of interest included glycemic control [including HbA1c, fasting plasma glucose (FBG)], weight loss, and cardiovascular risk factors [total cholesterol (TC), low-density lipoprotein cholesterol (LDL), high-density lipoprotein cholesterol (HDL), triacylglycerol (TG), diastolic blood pressure (DBP) and systolic blood pressure (SBP).

### Study selection

ENDNOTE X7 literature management software was used to manage the literature search records. To ensure high inter-rater reliability among the reviewers, a pilot-literature selection was performed.

Two reviewers independently screened the titles and abstracts of all the retrieved bibliographic records according to our eligibility criteria. Any studies with the potential to meet our inclusion criteria and conflicted studies were subjected to full-text evaluation. Any conflict was resolved by a third reviewer.

### Data extraction

Paired reviewers independently extracted the following data of interest: the first author, year of publication, country, study design (RCT), sample, diagnostic criteria for T2DM, study period, mean age, median weight, body mass index (BMI), mean baseline HbA1c, FBG, weight, LDL, HDL, TC, TG, SBP, and DBP, details of interventions, diabetes duration. Data were presented as the mean ± standard deviation (SD) at the end of the study; if values at the end of study were not available, they were imputed according to the Cochrane Handbook [27].

### Risk of bias of individual studies

The risk of bias in the included RCTs was assessed according to the Cochrane Handbook version 5.1.0 [27], including the method of adequate sequence generation, allocation concealment, blinding of participants and personnel, incomplete outcome data, selective reporting, and other sources of bias (e.g. early trial termination, extreme baseline imbalance). We classified the methodological quality as having a low, high, or unclear risk of bias. The risk of bias assessment was completed independently by two reviewers, and conflict was resolved by a third reviewer.

### Data analysis

We used the ‘*netmeta*’ version 0.9–2 of R-3.4.0 software to perform a Frequentist network meta-analysis [28]. The function of ‘*networkplot*’ function of STATA 15.1 (College Station, Texas 77,845 USA) was used to draw generate network plots to describe and present the geometry of different form of exercise. We used nodes to represent different interventions and edges to represent the head-to-head comparisons between interventions. The *‘decomp.design’* function was performed to assess the homogeneity in the whole network, the homogeneity within designs, and the homogeneity/consistency between designs. A node-splitting method was used to evaluate the inconsistency between direct and indirect comparisons [29]. Treatment ranking was calculated according to *P-scores*, which were based solely on the point estimates and standard errors of the network estimates. These scores measure the extent of certainty that a treatment is better than another treatment, averaged over all competing treatments [30].

A random effects network meta-analysis was performed to calculate pooled estimates and 95% confidence intervals (95%CI). In general, when the same measurement unit was used among studies for our outcomes of interest, the mean difference (MD) was considered as treatment effects to analyze the results or the standardized mean difference (SMD) was considered. However, when studies in meta-analyses were weighted by the inverse of the variance of the effect measure, the pooled SMD was associated with unfavorable negative bias. In our analysis, we used the ratio of the mean (RoM) to measure the treatment effect in the intervention group relative to that in the control group [31]; this value accounted for the baseline difference being roughly comparable through different measurement units. Then, we calculated and presented absolute risk differences (ARD) by using RoM and the baseline risk of no exercise.

We also planned to perform subgroup analyses to observe the discrepancy for a specific population. The subgroup factors were as follows: exercise length (longer term vs. shorter term, with 6 months used as a cut-off point based on the previous reviews) [32], type of patients (sedentary patients vs. non-sedentary patients, based on the reporting of the original studies), age (younger vs. older population, using age 60 years as a cut-off point based on World Health Organization report [33], and the duration of diabetes (longer duration vs. shorter duration, using the median duration of diabetes reported in the included studies as a cut-off point).

## Results

### Literature selection

**Fig. 1:**
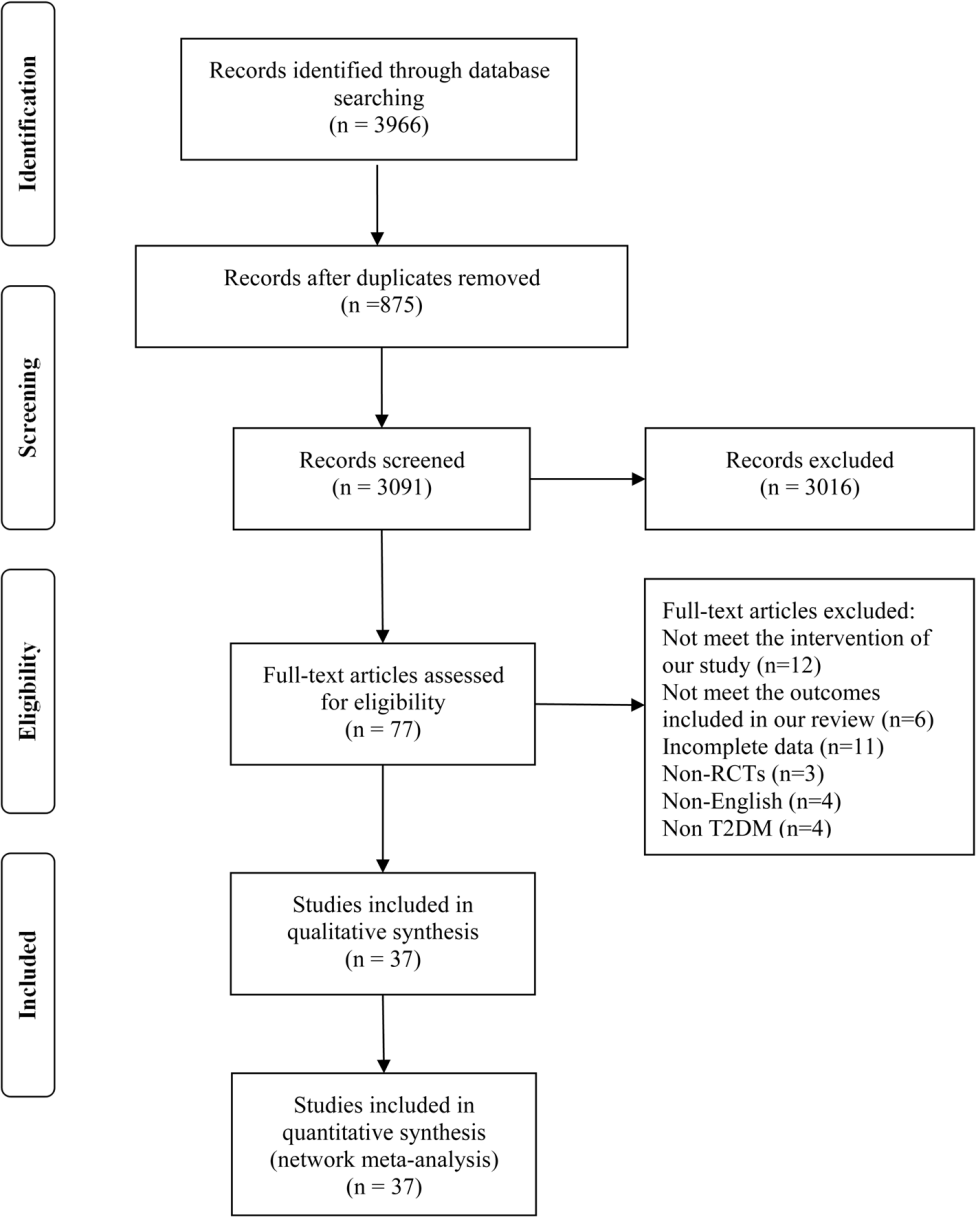
Flow Diagram

A total of 3966 studies were initially identified in this study. After reviewing the title and abstract, 75 studies were selected for further review. Of these, 41 were excluded (11 did not report the data of interest, 4 did not investigate T2DM, 3 were not RCTs, 12 did not meet our inclusion criteria, 6 did not report the outcomes included in our review, and 4 were non-English language). Finally, 37 studies met our inclusion criteria [34–70]. The detailed selection process is described in Fig. 1.

### Characteristics of the included studies

The characteristics of the included studies are presented in Table 1. A total of 2208 patients with T2DM were included into our network meta-analysis. The majority of the included studies consists of two arms and was published between 1998 and 2014. The study period of 26 included studies was less than 6 months. The mean age of patients ranged from 55.1 to 71.9 years. A total of 1079 patients enrolled in 14 studies reported previous sedentary behavior.

**Table 1:** Characteristic of included studies

Author	Country	Type of patients	Study period (months)	Interventions	Sample	Mean age (years)	Exercise prescription
					N/F/M		
Kwon 2010	Korea	NA	3	unsupervised RE	13/−/−	55.7 ± 6.2	40–50% 1RM; each session 40 min
				no exercise	15/−/−	57.0 ± 8.0	NA
Okada 2010	Japan	NA	3	combined	21/11/10	61.9 ± 8.6	3–5 times weekly; each session 60 min
				no exercise	17/6/11	64.5 ± 5.9	NA
Aylin 2009	Turkey	NA	2	combined	18/3/15	51.39 ± 2.02	RT: 50% 1RM; each session 30 min; AT: walking with moderate intensity
				no exercise	18/6/12	56.06 ± 1.48	NA
Kenneth 2013	Switzerland	Sedentary	3	supervised AE	20/−/−	68.5 ± 0.9	moderate to vigorous intensity; 3 times weekly; each session 60 min.
				anaerobic	20/−/−	70 ± 0.8	minimal aerobic component; nonstrenuous strength training
Larose 2011	Canada	Sedentary	6	combined	64/24/40	53.5 ± 7.3	NA
				supervised AE	60/21/39	53.9 ± 6.6	60% VO2peak; 75% heart rate; 3 times weekly
				supervised RE	64/24/40	54.7 ± 7.5	2–3 times weekly; each exercise 2–3 sets
				no exercise	63/22/41	54.8 ± 7.2	full aerobic training programme plus the full resistance training
Stolinskia 2008	UK	NA	6	supervised AE	8/4/4	59 ± 3	60–85%VO2peak; 4 times weekly; supervised by trainer
				unsupervised AE	9/4/5	55 ± 3	60–85%VO2peak; 4 times weekly Only contact trainers initially
Arslan 2014	Turkey	NA	3	supervised AE	31/14/17	53.5 ± 6.5	75% maximum heart rate; each session 45 min
				no exercise	33/19/14	54.0 ± 9.4	NA
Yavari 2010	Iran	NA	4	unsupervised AE	30/14/16	49.76 ± 6.56	60–85%VO2peak; 50% maximum heart rate;each session 4 min
				no exercise	30/18/12	49.86 ± 6.39	NA
Shenoy 2010	India	NA	2	unsupervised AE	20/5/15	53.15 ± 4.4	moderate intensity; 50–70% maximum heart rate; 150 min weekly
				no exercise	20/6/14	51 ± 5.4	NA
KU 2010	Korea	NA	3	supervised RE	13/13/0	55.7 ± 6.2	40–45% maximum capacity; 5 times weekly;
				supervised AE	15/15/0	55.7 ± 6.2	Moderate intensity; 5 times weekly;
				no exercise	16/16/0	57.8 ± 8.1	NA
Belli 2011	Brazil	Sedentary	3	supervised AE	9/9/0	55.9 ± 2.2	Time of walking increase from 20 min to 50 min
				no exercise	10/10/0	53.4 ± 2.3	NA
Dunstan 1998	Australia	Sedentary	2	supervised RE	11/3/8	50.3 ± 2.0	50–55% 1RM; each session 60 min;
				no exercise	10/5/5	51.1 ± 2.2	NA
Reid 2010	Canada	NA	6	combined	57/19/38	53.3 ± 7.2	full aerobic program plus the full resistance program
				supervised RE	58/20/38	54.7 ± 7.6	8 different exercises on weight machines each session; 3 times weekly
				supervised AE	51/18/33	53.8 ± 6.4	60–75% maximum heart rate; 3 times weekly
				no exercise	52/19/33	55.2 ± 6.9	NA
Sigal 2007	Canada	NA	6	combined	64/24/40	53.5 ± 7.3	full aerobic program plus the full resistance program
				supervised RE	60/21/30	53.9 ± 6.6	7 different exercises on weight machines each session
				supervised AE	64/24/40	54.7 ± 7.5	60–75% maximum heart rate; 3 times weekly
				no exercise	63/22/41	54.8 ± 7.2	NA
Choi 2012	Korea	Sedentary	3	no exercise	37/−/−	55.0 ± 6.0	NA
				unsupervised AE	38/−/−	53.8 ± 7.2	Moderate intensity; each session 60 min; 5 times weekly
Arora 2007	India	NA	2	no exercise	10/4/6	58.4 ± 1.8	NA
				supervised RE	10/6/4	49.6 ± 5.2	60% 1RM; 3 sets of 10 repetitions of 7 exercise each session;
				unsupervised AE	10/4/6	52.2 ± 9.3	2 times weekly; each session 30 min
Oliveira 2012	Brazi	NA	3	flexibility training	12/8/4	53.42 ± 9.82	perform stretching exercises
				supervised AE	11/6/5	52.09 ± 8.71	Cycling time increase from 20 min to 50 min
				supervised RE	10/6/4	54.10 ± 8.94	50% 1RM; 4 sets of 8 to 12 repetitions of 7 exercise
				combined	10/6/4	57.90 ± 9.82	same intensity and half the volume of that in the AT and ST groups
Jennings 2009	Canada	Sedentary	6	combined	19/6/13	54.48 ± 7.68	3 times weekly
				supervised RE	18/7/11	52.84 ± 7.54	3 times weekly
				supervised AE	13/7/6	55.35 ± 7.49	3 times weekly
				no exercise	22/11/11	56.33 ± 6.91	NA
Cauza 2005	Australia	NA	4	unsupervised RE	22/11/11	56.4 ± 1.1	3 sets per muscle group per week; 3 times weekly
				unsupervised AE	17/8/9	57.9 ± 1.4	60% VO_2max_; 3 times weekly
Franciele 2013	USA	NA	7 days	unsupervised AE	7/2/5	56 ± 2	70% peak heart rate; each session 40 min
				combined	7/2/5		20 min AT at 70% peak heart rate and 4 RT 3 sets of 12 repetitions at 65% 1RM
Cheung 2009	Australia	Sedentary	4	unsupervised RE	20/13/7	59 ± 8.7	5 times weekly; each session 30 min
				no exercise	17/12/5	62 ± 6.7	NA
Whye 2011	Singapore	Sedentary	2	unsupervised RE	30/19/11	57 ± 7	65% maximum heart rate; 9 resistive exercises 3 sets of 10 repetitions
				unsupervised AE	30/22/8	59 ± 7	50 min AT exercise; 65% maximum heart rate
Morton 2012	UK	NA	1.65	supervised AE	27/6/21	61 ± 10	Walking in duration from 25 to 55 min.
				no exercise	27/6/21	63 ± 9	NA
Dede 2014	Turkey	Sedentary	3	supervised AE	30/15/15	52.5 ± 7.5	60–75% maximum heart rate
				no exercise	30/16/14	55.5 ± 8.4	NA
BACCHI 2012	Italy	NA	4	supervised AE	20/6/14	57.2 ± 1.6	60–65% maximum heart rate
				supervised RE	20/6/14	55.6 ± 1.7	30–50%1 RM; three series of 10 repetitions
Ng 2010	Singapore	Sedentary	2	supervised AE	30/11/19	57 ± 7	NA
				supervised RE	30/8/22	59 ± 7	65% 1RM;
Sparks 2013	Netherlands	Sedentary	9	flexibility training	10/8/2	60.8 ± 8.0	65% maximum heart rate
				supervised AE	12/6/6	54.2 ± 6.0	50–80% VO_2_ peak;
				supervised RE	18/9/9	60.4 ± 7.3	Each session 45–50 min; each set consisted of 10 to 12 repetitions
				combined	12/6/6	54.1 ± 6.2	NA
Gavin 2010	Canada	Sedentary	6	no exercise	63/22/41	54.8 ± 7.2	NA
				supervised AE	60/21/39	53.9 ± 6.6	65% maximum heart rate; 50% of VO_2_peak
				supervised RE	64/24/40	54.7 ± 7.5	2 to 3 times weekly; maximum of 8 repetitions
				combined	64/24/40	53.5 ± 7.3	full Aerobic program plus the full Resistance program
Madden 2009	Canada	NA	3	supervised AE	18/−/−	71.7 ± 1.1	Each session 60 min; 60–75% maximum heart rate
				An-AE	18/−/−	71.1 ± 0.9	3 times weekly; no aerobic component and consisted of nonaerobic core and dumbbells exercises
Madden 2011	Canada	Sedentary	3	supervised AE	21/15/25	71.9 ± 1.1	3 times weekly; each session 60 min
				An-AE		71.3 ± 0.9	3 times weekly; no aerobic component and consisted of nonaerobic core and dumbbells exercises
William 2011	New Zealand	NA	4	supervised RE	9/6/3	48 ± 6	two to three sets of eight major exercises
				supervised AE	9/7/2	51 ± 4	3 times weekly; each session 40–60 min;
Kadoglou 2014	Greece	Sedentary	6	supervised AE	30/17/13	59.33 ± 4.76	50–75%, VO_2_ peak; each session 60 min
				no exercise	30/18/12	63.82 ± 7.03	NA
ALAM 2004	UK	NA	6	supervised AE	9/5/4	59.5 ± 2.5	60–85%, VO_2_ peak; supervised by trainer
				unsupervised AE	9/4/5	55.3 ± 23.2	NA
Tessier 2000	Canada	NA	3	supervised AE	19/7/12	69.3 ± 4.2	60–79% maximum heart rate
				no exercise	20/9/11	69.5 ± 5.1	NA
Church 2011	American	Sedentary	3	no exercise	41/28/13	58.6 ± 8.2	NA
				supervised RE	73/43/30	56.9 ± 8.7	3 times per week; each set consisted of 10 to 12 repetitions
				supervised AE	72/45/27	53.7 ± 9.1	50–80% maximum heart rate; 150 min per week
				Combined	76/49/27	55.4 ± 8.3	NA
Kwon 2010b	Korea	NA	6	unsupervised AE	13/13/0	55.5 ± 7.5	5 times per week;
				no exercise	14/14/0	57.5 ± 8.6	NA
Winnick 2008	USA	NA	4	unsupervised AE	15 (the white)	49.5 ± 2.9	10 repetition performed on each of eight machines
				unsupervised RE	18 (the white)	50.3 ± 3.5	3 times weekly; each session 30-40mijn
				unsupervised AE	24 (African)	50.7 ± 2.0	NA
				unsupervised RT	12 (African)	46.2 ± 2.0	NA

NOTE: AE: aerobic exercise; RE: resistance exercise; NA: not available; RM: repetition maximum

**Fig. 2:**
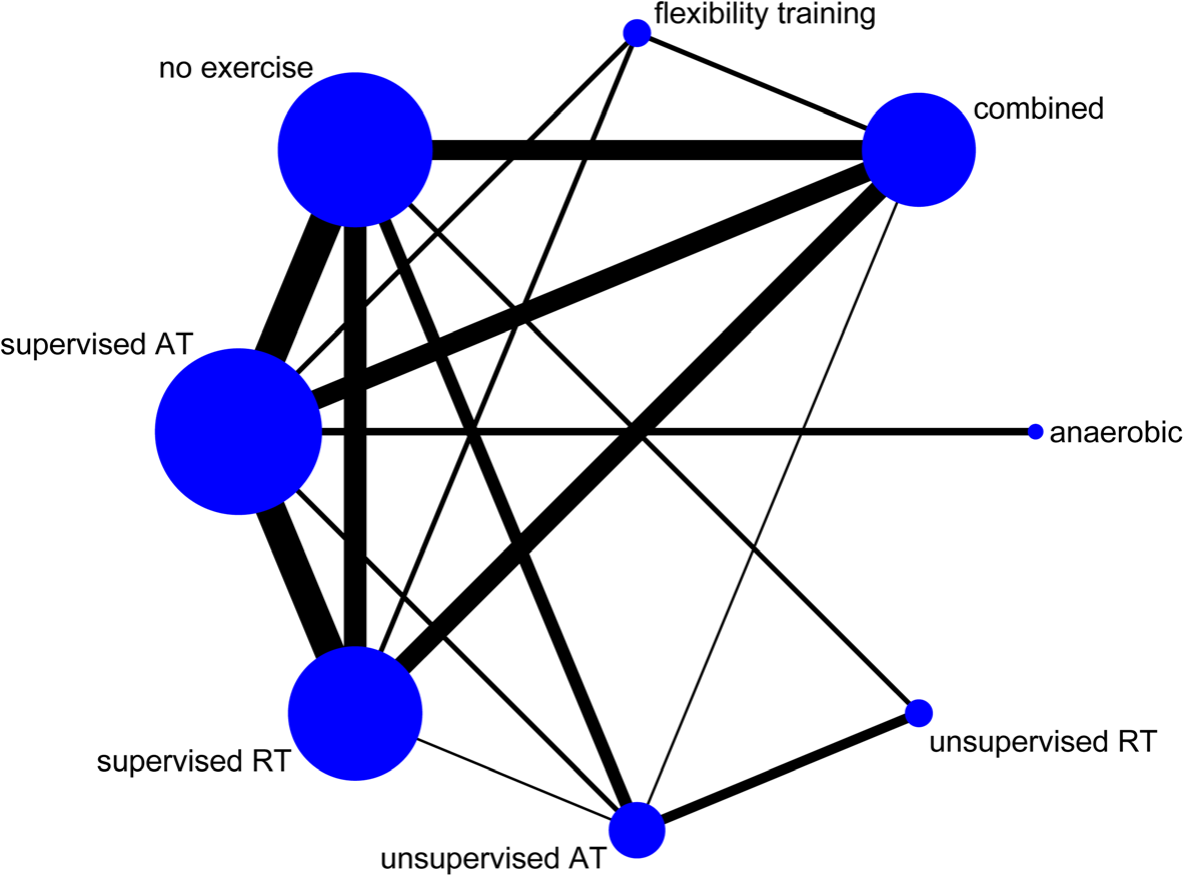
network plot

We included the following eight exercise training modalities in our network meta-analysis (Fig. 2): supervised aerobic, unsupervised aerobic, anaerobic, supervised resistance, unsupervised resistance, combined exercise, flexibility training, and no exercise.

### Results of risk of bias

The results of risk of bias are provided in Additional file 1: Appendix 3. Two articles were judged to have a high risk of bias in adequate sequence generation [40, 66]. Fifteen articles showed unclear risk of bias [37–39, 41, 42, 47, 50, 55, 57, 58 63–65, 67, 68] and six showed high risk of bias [40, 51, 52, 61, 62, 66] in adequate allocation concealment. Blinded outcome assessment was reported in 10 studies [39, 44–47, 49, 53, 59, 60, 69]. In terms of incomplete outcome data, four studies were judged as high risk [36, 54, 66, 69] and three were unclear risk [35, 43, 48, 67]. Eleven studies showed unclear risk of bias in selective reporting [34, 35, 40, 41, 45, 47, 50, 53, 59, 60, 66], and 16 showed unclear risks of other biases [38, 42, 44, 45, 47, 48, 50, 55, 57, 59, 60, 62, 63, 65, 67, 69].

### Network meta-analysis

#### Glycemic control

Twenty-six studies involving 1729 patients reported HbA1c and FBG. Of these, two studies [64, 65] were not included in our network meta-analysis of HbA1c and our study [43] was not included in our network meta-analysis of FBG due to the imbalance in the baselines in these studies.

Compared to no exercise, combined exercise (− 0.53, 95%CI: -0.68% to − 0.45%), supervised aerobic (− 0.30, 95%CI: -0.60% to − 0.45%), supervised resistance (− 0.30, 95%CI: -0.38% to − 0.15%), and flexibility training (− 0.60, 95%CI: -1.05% to − 0.15%) showed significant reduction in HbA1c (Table 2, Additional file 1: Appendix 6). Compared to unsupervised aerobic and unsupervised resistance exercises, supervised aerobic (− 0.60, 95%CI: -0.83% to − 0.30%; − 0.60, 95%CI: -0.83% to − 0.20%; respectively) and supervised resistance (− 0.53% lower, 95%CI: -0.75% to − 0.30%; − 0.53, 95%CI: -0.83% to − 0.23%; respectively) exercises showed more benefit in reducing HbA1c (Table 2, Additional file 1: Appendix 6). Combined exercise resulted in the most significant reduction in HbA1c when compared with supervised aerobic (− 0.23, 95%CI: -0.30% to − 0.08%), unsupervised aerobic (− 0.75, 95%CI: -0.98% to − 0.53%), supervised resistance (− 0.23, 95%CI: -0.38% to − 0.15%), and unsupervised resistance (− 0.75, 95%CI: -0.98% to − 0.45%) exercises (Table 2, Additional file 1: Appendix 6). Furthermore, combined exercise also showed the greatest potential as the best intervention to improve HbA1c (*P*-*score* = 0.99, Additional file 1: Appendix 6). However, there were no significant differences between the effectiveness of the other exercises in reducing HbA1c levels (Table 2).

**Table 2:** Results of network meta-analysis

Comparisons	Network meta-analysis RoM [95%CI]								
	HbA1c	FBG	weight	TC	LDL	HDL	TG	SBP	DBP
No exercise (reference)									
Supervised aerobic	0.96 [0.94; 0.97]	0.93 [0.88; 0.98]	0.97 [0.94; 1.00]	0.89 [0.85; 0.94]	0.89 [0.80; 0.99]	0.92 [0.89; 0.96]	0.87 [0.80; 0.96]	0.97 [0.95 1.00]	0.97 [0.94; 1.00]
Supervised resistance	0.96 [0.95; 0.97]	1.01 [0.92; 1.12]	0.98 [0.94; 1.01]	0.88 [0.83; 0.94]	0.88 [0.76; 1.01]	0.90 [0.85; 0.94]	0.89 [0.81; 0.97]	0.96 [0.93; 0.99]	0.97 [0.93; 1.00]
Unsupervised aerobic	1.03 [1.00; 1.07]	1.11 [0.81; 1.52]	1.04 [1.00; 1.08]	0.96 [0.90; 1.02]	1.08 [0.88; 1.33]	0.99 [0.87; 1.12]	0.95 [0.86; 1.05]	0.97 [0.93; 1.01]	0.94 [0.89; 1.00]
Unsupervised resistance	1.03 [0.99; 1.07]	1.03 [0.72; 1.47]	0.97 [0.91; 1.02]	–	1.12 [0.81; 1.56]	0.91 [0.71; 1.17]	–	0.95 [0.87; 1.04]	0.97 [0.86; 1.09]
Anaerobic (reference)									
Combined	–	0.98 [0.84; 1.15]	1.00 [0.91; 1.11]	–	–	–	–	0.98 [0.89; 1.08]	0.95 [0.86; 1.04]
No exercise	–	1.05 [0.97; 1.14]	1.07 [0.98; 1.17]	–	–	–	–	1.01 [0.92; 1.11]	0.96 [0.88; 1.04]
Supervised aerobic	–	0.98 [0.92; 1.03]	1.05 [0.97; 1.14]	–	–	–	–	0.99 [0.90; 1.07]	0.93 [0.86; 1.00]
Supervised resistance	–	1.07 [0.96; 1.19]	1.05 [0.96; 1.15]	–	–	–	–	0.97 [0.89; 1.07]	0.93 [0.85; 1.01]
Unsupervised aerobic	–	1.17 [0.86; 1.60]	1.12 [1.02; 1.22]	–	–	–	–	0.98 [0.89; 1.08]	0.91 [0.82; 1.00]
Unsupervised resistance	–	1.08 [0.76; 1.54]	1.04 [0.93; 1.15]	–	–	–	–	0.96 [0.85; 1.09]	0.93 [0.81; 1.07]
Combined (reference)									
Flexibility	1.15 [0.98; 1.34]	–	–	0.92 [0.84; 1.02]	0.96 [0.78; 1.19]	0.97 [0.89; 1.05]	–	–	–
No exercise	1.08 [1.06; 1.09]	1.07 [0.94; 1.22]	1.07 [1.01; 1.13]	0.97 [0.92; 1.03]	1.08 [0.95; 1.23]	1.03 [1.00; 1.07]	1.34 [1.19; 1.50]	1.03 [0.99; 1.08]	1.01 [0.96; 1.06]
Supervised aerobic	1.03 [1.02; 1.05]	0.99 [0.86; 1.15]	1.05 [0.99; 1.11]	0.87 [0.82; 0.93]	0.96 [0.85; 1.10]	0.95 [0.91; 0.99]	1.17 [1.03; 1.33]	1.01 [0.97; 1.05]	0.98 [0.93; 1.03]
Supervised resistance	1.03 [1.02; 1.05]	1.09 [0.92; 1.28]	1.05 [0.99; 1.11]	0.86 [0.80; 0.92]	0.95 [0.82; 1.10]	0.92 [0.88; 0.97]	1.18 [1.04; 1.35]	0.99 [0.95; 1.04]	0.98 [0.92; 1.03]
Unsupervised aerobic	1.11 [1.07; 1.15]	1.19 [0.85; 1.68]	1.11 [1.04; 1.19]	0.93 [0.86; 1.01]	1.17 [0.93; 1.47]	1.01 [0.93; 1.11]	1.27 [1.10; 1.47]	1.00 [0.95; 1.06]	0.95 [0.89; 1.03]
Unsupervised resistance	1.11 [1.06; 1.16]	1.10 [0.76; 1.61]	1.03 [0.95; 1.12]	–	1.22 [0.86; 1.72]	0.93 [0.79; 1.11]	–	0.98 [0.89; 1.09]	0.98 [0.86; 1.11]
Flexibility (reference)									
No exercise	0.94 [0.80; 1.09]	–	–	1.05 [0.95; 1.17]	1.13 [0.91; 1.41]	1.06 [0.98; 1.16]	–	–	–
Supervised aerobic	0.89 [0.76; 1.05]	–	–	0.92 [0.81; 1.04]	1.00 [0.81; 1.24]	0.98 [0.90; 1.06]	–	–	–
Supervised resistance	0.90 [0.77; 1.05]	–	–	0.94 [0.83; 1.07]	0.99 [0.80; 1.22]	0.95 [0.88; 1.03]	–	–	–
Unsupervised aerobic	0.97 [0.83; 1.14]	–	–	1.01 [0.90; 1.13]	1.21 [0.94; 1.57]	1.04 [0.93; 1.17]	–	–	–
Unsupervised resistance	0.96 [0.82; 1.13]	–	–	–	1.27 [0.86; 1.86]	0.96 [0.80; 1.16]	–	–	–
Supervised aerobic (reference)									
Supervised resistance	1.01 [0.99; 1.02]	1.09 [1.00; 1.20]	1.01 [0.97; 1.05]	0.99 [0.93; 1.05]	0.99 [0.86; 1.12]	0.98 [0.90; 1.06]	1.01 [0.91; 1.14]	0.99 [0.96; 1.02]	1.00 [0.96; 1.04]
Unsupervised aerobic	1.08 [1.05; 1.12]	1.20 [0.88; 1.63]	1.07 [1.03; 1.11]	1.07 [1.00; 1.15]	1.21 [0.99; 1.48]	1.02 [0.89; 1.17]	1.09 [0.96; 1.24]	0.99 [0.95; 1.04]	0.97 [0.92; 1.04]
Unsupervised resistance	1.08 [1.03; 1.12]	1.11 [0.78; 1.57]	1.00 [0.94; 1.06]	–	1.26 [0.91; 1.75]	1.26 [0.95; 1.67]	–	0.98 [0.89; 1.07]	0.99 [0.91; 1.08]
Supervised resistance (reference)									
Unsupervised aerobic	1.08 [1.04; 1.12]	1.10 [0.80; 1.51]	1.06 [1.01; 1.11]	1.09 [1.01; 1.16]	1.23 [0.98; 1.56]	1.05 [0.91; 1.20]	1.08 [0.97; 1.19]	1.01 [0.96; 1.05]	0.98 [0.92; 1.04]
Unsupervised resistance	1.07 [1.03; 1.12]	1.01 [0.71; 1.45]	0.99 [0.92; 1.06]	–	1.28 [0.90; 1.81]	0.96 [0.75; 1.25]	–	0.99 [0.90; 1.09]	1.00 [0.89; 1.13]
Unsupervised aerobic (reference)									
Unsupervised resistance	1.00 [0.97; 1.03]	0.92 [0.79; 1.09]	0.93 [0.87; 0.99]	–	1.04 [0.80; 1.35]	0.92 [0.74; 1.14]	–	0.98 [0.91; 1.07]	1.03 [0.93; 1.14]

**NOTE:** Comparison: Treatment comparison; RoM: ratio of mean; FBG: fasting plasma glucose; SBP: systolic blood pressure; DBP: diastolic blood pressure; TC: total cholesterol; TG: triacylglycerol; LDL: low-density lipoprotein cholesterol; HDL: high-density lipoprotein cholesterol; −: results not reported in studies

Subgroup analysis (Additional file 1: Appendix 5) showed that supervised aerobic and supervised resistance forms of exercise were not significantly better than unsupervised aerobic exercise in reducing HbA1c levels when the study duration was less than 6 months (*P*_interaction_ < 0.05). Significant interaction discrepancies were not found in our analyses of other subgroups.

Similar to the effects on HbA1c, supervised aerobic significantly reduced FBG by 9.38 mg/dl (Table 2, Additional file 1: Appendix 4), and ranking probability showed that supervised aerobic exercise had the most significant ability to reduce FBG (*P-score* = 0.82, Additional file 1: Appendix 6). Subgroup analyses did not show significant interaction discrepancy between any of the subgroup factors (Additional file 1: Appendix 5).

#### Weight loss

Seventeen studies involving 662 patients reported weight loss and were included in our network meta-analysis.

Compared to unsupervised aerobic exercise, combined (− 8.37 kg, 95%CI: -13.39 kg to − 3.35 kg), supervised aerobic (− 5.02 kg, 95%CI: -8.37 kg to − 1.67 kg), supervised resistance (− 5.02 kg, 95%CI: -9.21 kg to − 0.84 kg), and anaerobic (− 8.37 kg, 95%CI: -15.07 kg to − 1.67 kg) forms of exercise showed greater weight reduction (Table 2, Additional file 1: Appendix 6). Subgroup analysis showed that these differences tended to be greater in studies of longer duration (Additional file 1: Appendix 5).

Combined exercise also showed more benefit in terms of weight loss compared to the effects of no exercise (− 5.02 kg, 95%CI: -9.21 kg to − 0.84 kg). In addition, combined exercise showed the most significant effectiveness in terms of weight loss (*P -score* = 0.86, Additional file 1: Appendix 6).

#### Cardiovascular risk factors

Twenty-two studies involving 1323 patients reported SBP, DBP, TC, TG, LDL and HDL. Of these, one study [68] was not included in our network meta-analysis of TC due to imbalance in the baseline.

Compared to no exercise, supervised aerobic (TC: − 20.24 mg/dl, 95%CI: -27.60 mg/dl to − 11.04 mg/dl; TG: − 19.34 mg/dl, 95%CI: -29.76 mg/dl to − 5.95 mg/dl; LDL: − 11.88 mg/dl, 95%CI: -21.60 mg/dl to − 1.08 mg/dl; HDL: − 3.66 mg/dl, 95%CI: -5.04 mg/dl to − 1.83 mg/dl), supervised resistance (SBP: − 5.20 mmHg, 95%CI: -9.10 mmHg to − 1.30 mmHg; TC: − 22.08 mg/dl, 95%CI: -31.28 mg/dl to − 11.04 mg/dl; TG: − 16.37 mg/dl, 95%CI: -28.27 mg/dl to − 4.46 mg/dl; HDL: − 4.58 mg/dl, 95%CI: -6.87 mg/dl to 2.75 mg/dl) and combined (TG: − 37.20 mg/dl, 95%CI: -49.10 mg/dl to − 23.81 mg/dl) exercises showed better improvement in SBP, TC, TG and LDL (Table 2, Additional file 1: Appendix 6).

Supervised aerobic (− 23.92 mg/dl, 95%CI: -33.12 mg/dl to − 12.88 mg/dl) and supervised resistance (− 25.76 mg/dl, 95%CI: -36.80 mg/dl to − 14.72 mg/dl) exercise showed greater improvement in TC and HDL compared with the effects of combined exercise, while combined exercise induced a greater reduction in TG than supervised aerobic (− 25.76 mg/dl, 95%CI: -46.00 mg/dl to − 3.68 mg/dl) and supervised resistance (− 29.44 mg/dl, 95%CI: -47.84 mg/dl to − 7.36 mg/dl) exercises (Table 2).

Supervised resistance showed the most significant improvements in HDL, LDL and TC (*P-score* = 0.74, 0.79, 0.92, respectively, Additional file 1: Appendix 6), and combined exercise showed the most significant improvements in TG (*P-score* = 0.99).

Subgroup analyses showed that supervised aerobic exercise was associated with a greater reduction in SBP than unsupervised aerobic exercise in the older population (*P*_interaction_ < 0.05), a greater reduction in LDL than combined exercise in the older population (*P*_interaction_ < 0.05), and a greater reduction in HDL than supervised resistance exercise with longer disease duration (*P*_interaction_ < 0.05) (Additional file 1: Appendix 5).

### Inconsistency between direct and indirect comparisons

Assessment of inconsistency between direct and indirect comparisons using a node-splitting model showed that there were no inconsistencies among most studies (*P* > 0.05) (Additional file 1: Appendix 4).

## Discussion

In our study, we used both direct and indirect evidence to evaluate the relative effects of different exercises on glycemic control, cardiovascular risk factors, and weight loss in patients with T2DM. In particular, we separated the differences between supervised and unsupervised forms of exercise. Integrating the currently available data, our network meta-analysis indicated that combined exercise was as effective in reducing HbA1c as supervised aerobic and supervised resistance forms of exercise. Supervised aerobic training and supervised resistance training were more powerful in improving SBP, TC, and HDL than combined exercise. *P-Score* ranking revealed that combined, supervised aerobic, and supervised resistance forms as the top three exercise modalities. Moreover, supervised exercise showed more benefit than unsupervised exercise for most comparison groups.

Our study suggested those who wished to improve their HbA1c through lifestyle management to engage in combined exercise. Recently, increasing importance has been attached to the effect of combined exercise. One study [49] showed that combined exercise was more effective than either aerobic or resistance training alone in reducing HbA1c, which was consistent with the findings of our analysis. Therefore, Appropriate exercise is recommended for T2DM patients as a part of their therapy, although this approach is usually unsuccessful unless the training is supervised. It could be speculated that this was because therapeutic exercise strategies that were designed by professional trainers were often ineffective in improving the adverse lipid profile and decreasing insulin resistance. Evidence from RCTs showed a decrease in HbA1c, fasting insulin concentration, and FBG in the supervised aerobic group, but not in the corresponding unsupervised group [63]. The results of our study also showed that supervised aerobic/resistance could effectively manage patients with T2DM than unsupervised group.

Onset of T2DM, cardiovascular disease and even cardiovascular mortality are inversely related to cardiorespiratory fitness [71]. Compared with the non-diabetic population, the population with T2DM is associated with a higher risk of cardiovascular disease (2 to 4-fold increase) [72]. Regular aerobic exercise can increase insulin sensitivity, which improves the adverse lipid profile [73, 74]. It has been shown that resistance exercise can benefit all adults and patients with T2DM by improving physical function, fat mass, lipid profiles, cardiovascular health, blood pressure, and insulin sensitivity [75, 76]. Meta-analyses performed by Kelley [77] showed that aerobic exercise was more efficient for lowering LDL in patients with T2DM than other types of training. However, the meta-analyses by Kelley failed to show the comparative effectiveness of aerobic exercise for improving other cardiovascular risk factors. Our meta-analysis showed that both supervised aerobic and supervised resistance exercise had the similar effect in improving LDL and TC. Thus, it may be significantly helpful for clinicians, policy makers, and patients with T2DM to recommend either form of exercise for the prevention of cardiovascular disease.

Schwingshackl [32] compared the effects of different training modalities on glycemic control and blood lipids in patients with T2DM. The objective of their study was similar to that of ours. However, we conducted a more comprehensive analysis in that we included not only supervised aerobic exercise and supervised resistance exercise, but also unsupervised aerobic and unsupervised resistance exercise, as well as no exercise, and flexibility training. In addition, we focused on more outcomes including glycemic control, cardiovascular risk factors, and weight loss. Importantly, because the different units of measurement for all outcomes of interest were used in included RCTs, we usually analyzed a pool of these studies using SMD. However, since it is difficult to explain this parameter to evidence users, we employed the RoM to measure the relative effect differences between the intervention and control groups. Finally we calculated absolute effect differences using no exercise as the baseline risk, which is more straightforward and more understandable to evidence users.

To evaluate the evidence in a specific population, we performed four subgroup analyses in term of study duration, diabetes duration, age, and type of population. We found that supervised aerobic exercise and supervised resistance exercise showed more benefit in reducing HbA1c than unsupervised aerobic exercise only when the study duration was longer than 6 months. Supervised aerobic exercise showed a greater reduction in SBP than unsupervised aerobic exercise in the older population, and a greater reduction in HDL than supervised resistance for patients with longer disease duration. No significant interaction discrepancies in most of the outcomes were found for most of the comparison groups.

Some limitations of our study should be noted. First, we used RoM (post-intervention/post-control) to account for the changes from baseline and some RCTs showed significant differences in the baseline; therefore, those RCTs were not included in our final network meta-analysis model. Second, previous dose-response meta-regression analysis [78] revealed that the reduction in HbA1c was associated with exercise frequency for supervised aerobic exercise, and associated with weekly volume of resistance for supervised combined exercise although further studies are required to confirm these associations. Finally, we planned to include quality of life as a primary outcome in our study protocol; however, the scales for measurement of quality of life were widely inconsistent. For example, Holton’s study [79] used a 36-item Short-Form Health Survey, while Bello’s study [80] used the WHO Quality of Life questionnaire, and Fritz’s study [81] used the Swedish Health-Related Quality of Life questionnaire. Our network meta-analysis included eight interventions and nine outcomes with extremely complex networks; therefore, we consider that quality of life should be evaluated in a separate study.

## Conclusions

Combined exercise showed more pronounced improvement in HbA1c than either supervised aerobic exercise or supervised resistance exercise alone; however, the decrease in some cardiovascular risk factors was less marked. In terms of weight loss, there were no significant differences among the combined, supervised aerobic and supervised resistance forms of exercise.

## Additional file

Additional file 1: Appendix 1 Search strategies. Appendix 2 Definition of interventions. Appendix 3 Results of risk of bias. Appendix 4 Results of direct, indirect, network meta-analyses, and inconsistency. Appendix 5 subgroup analyses. Appendix 6 Absolute effect estimates of different exercise modalities using no exercise as baseline risk (PDF 400 kb)

## Abbreviations

ACSM: American College of Sports Medicine
ADA: American Diabetes Association
BPTA: Belgian Physical Therapy Association
combined exercise: combined aerobic exercise and resistance exercise
DBP: diastolic blood pressure
ESC: European Society of Cardiology
ESSA: Exercise and Sports Science Australia
FBG: fasting plasma glucose
HDL: high-density lipoprotein cholesterol
LDL: low-density lipoprotein cholesterol
MD: mean difference
RCT: randomized controlled trials
RoM: ratio of mean
SBP: systolic blood pressure
SMD: standardized mean difference
SNIPH: Swedish National Institute of Public Health
T2DM: Type 2 diabetes
TC: total cholesterol
TG: triacylglycerol
