# Supplementary material for: Exercise training modalities in patients with type 2 diabetes mellitus: a systematic review and network meta-analysis
Source: Int J Behav Nutr Phys Act. 2018 Jul 25;15:72. doi: 10.1186/s12966-018-0703-3 (PMC6060544; doi:10.1186/s12966-018-0703-3)
Supplement: Supplementary file 1 — Appendix 1 Search strategies. Appendix 2 Definition of interventions. Appendix 3 Results of risk of bias. Appendix 4 Results of direct, indirect, network meta-analyses, and inconsistency. Appendix 5 subgroup analyses. Appendix 6 Absolute effect estimates of different exercise modalities using no exercise as baseline risk (PDF 400 kb) [file 12966_2018_703_MOESM1_ESM.pdf]

Exercise training modalities in patients with type 2 diabetes mellitus: a systematic review and network meta-analysis

Bei Pan [1#], Long Ge [1,2,3#], Yang-qin Xun [1,3], Ya-jing Chen[1], Cai-yun Gao [1], Xue Han [1], Li-qian Zuo[1], Ke-hu Yang [3], Jin-hui Tian [3*], Guo-wu Ding [1]

1 Department of Social Medicine and Health Management, School of Public Health, Lanzhou University, Lanzhou 730000, China

2 The First Clinical Medical College, Lanzhou University, Lanzhou 730000, China

3 Evidence-Based Medicine Center, School of Basic Medical Sciences, Lanzhou University, Lanzhou 730000, China

Contents

Appendix 1 Search strategies 3

Appendix 2 Definition of interventions 5

Appendix 3 Results of Results of risk of bias 6

Appendix 4 Results of direct, indirect, and network meta-analyses 8

Appendix 4-1 Results of network meta-analyses on HbA1c 8

Appendix 4-2 Results of network meta-analyses on FBG 10

Appendix 4-3 Results of network meta-analyses weight 12

Appendix 4-4 Results of network meta-analyses on SBP 14

Appendix 4-5 Results of network meta-analyses on DBP 16

Appendix 4-6 Results of network meta-analyses on TC 18

Appendix 4-7 Results of network meta-analyses on TG 20

Appendix 4-8 Results of network meta-analyses on LDL 22

Appendix 4-9 Results of network meta-analyses on HDL 24

Appendix 5 Subgroup analyses 26

Appendix 6 Absolute effect estimates of different exercise modalities using no exercise as baseline risk 62

Appendix 1 Search strategies
PubMed:

(((((("Diabetes Mellitus, Type 2"[Mesh]) OR "Diabetes Mellitus"[Title/Abstract]) OR NIDDM[Title/Abstract] OR MODY[Title/Abstract] OR T2DM[Title/Abstract] OR T2D[Title/Abstract] OR "non-insulin* depend*"[Title/Abstract] OR "noninsulin* depend*"[Title/Abstract] OR "non insulindepend*"[Title/Abstract] OR noninsulindepend*[Title/Abstract] AND (("Exercise Therapy"[Mesh]) OR (("muscle strength"[Title/Abstract] OR "muscle strengthening"[Title/Abstract] OR "muscle-strengthening"[Title/Abstract] OR "weight lifting"[Title/Abstract] OR weight-lifting[Title/Abstract] OR "weight bearing"[Title/Abstract] OR weight-bearing[Title/Abstract] OR "weight training"[Title/Abstract] OR "circuit training"[Title/Abstract] OR "strength exercise"[Title/Abstract] OR "strengthening exercise"[Title/Abstract] OR "strength training"[Title/Abstract] OR "resistance exercise"[Title/Abstract] OR "resistance training"[Title/Abstract] OR "progressive resistance"[Title/Abstract] OR "Physical Exercise"[Title/Abstract] OR "Isometric Exercise"[Title/Abstract] OR "aerobic exercise"[Title/Abstract] OR "aerobic training"[Title/Abstract] OR "exercise therapy"[Title/Abstract])))))) AND random*

Cochrane Central Register of Controlled Trials :

#1 "Diabetes Mellitus" or "Diabetes Insipidus" or NIDDM or T2DM or T2D or "non insulin* depend*" or "noninsulin* depend*" or "non insulindepend*" or "noninsulindepend*" :ti,ab,kw (Word variations have been searched)

#2 MeSH descriptor: [Diabetes Mellitus] explode all trees

#3 #1 OR #2

#4 "muscle strength" or "muscle strengthening" or "muscle-strengthening" or "weight lifting" or weight-lifting or "weight bearing" or weight-bearing or "weight training" or "circuit training" or "strength exercise" or "strengthening exercise" or "strength training" or "resistance exercise" or "resistance training" or "progressive resistance" or "Physical Exercise" or "Isometric Exercise" or "aerobic exercise" or "aerobic training" or "exercise therapy" or "diet*":ti,ab,kw (Word variations have been searched)

#5 MeSH descriptor: [Exercise Therapy] explode all trees

#6 #4 OR #5

#7 #3 AND #6

EMBASE:

#1 'muscle strength'/exp OR 'muscle strength' OR 'muscle strengthening'/exp OR 'muscle strengthening' OR 'muscle-strengthening'/exp OR 'muscle-strengthening' OR 'weight lifting'/exp OR 'weight lifting' OR 'weight bearing'/exp OR 'weight bearing' OR 'weight training' OR 'circuit training'/exp OR 'circuit training' OR 'strength exercise' OR 'strengthening exercise' OR 'strength training'/exp OR 'strength training' OR 'resistance exercise'/exp OR 'resistance exercise' OR 'resistance training'/exp OR 'resistance training' OR 'progressive resistance' OR 'physical exercise'/exp OR 'physical exercise' OR 'isometric exercise'/exp OR 'isometric exercise' OR 'aerobic exercise'/exp OR 'aerobic exercise' OR 'aerobic training' OR 'exercise therapy'/exp OR 'exercise therapy'

#2 'diabetes mellitus'/exp OR 'diabetes mellitus' OR 'niddm'/exp OR niddm OR t2dm OR t2d OR non AND noninsulin* OR non insulindepend* OR noninsulindepend*

#3 #1 AND #2

Appendix 2 Definition of interventions

Aerobic training (AT): We defined AT as a regimen containing aerobic components performed at least three to five times per week for at least four weeks and performing minimum for 30mins each time. Aerobic components included walking, cycling, jogging, and swimming but not limit to these types [1].

Resistance training (RT): We defined RT as exercise performed against some type of progressive resistance on a minimum of two days each week to increase their muscle strength, muscle endurance or muscle power [2]. Resistance components included bench press, seated row, shoulder press, leg press, and weight strength but not limit to these types.

Anaerobic training (an-AT): We defined anaerobic training as high intensity and instantaneous exercise (VO_2max is low), as well as have no aerobic components and consisted of nonaerobic core (exercise ball).

Supervised aerobic training: We defined supervised aerobic training as participants done the aerobic exercise and supervised by trainers.

Unsupervised aerobic training: People participated in home-based aerobic exercise or the RCTs did not mention whether people in this group were supervised by trainers.

Supervised resistance training: Participant done the resistance exercise and supervised by trainers.

Unsupervised resistance training: People participated in home-based resistance exercise or the RCTs did not mention whether people in this group were supervised by trainers.

Combined aerobic training and resistance training (combined exercise): Participant performed the aerobic training program plus the resistance training program to assure an adequate dose of each type of exercise.

Flexibility training: Participants were offered weekly stretching and relaxation classes.

No exercise: Participant were asked do not to participant in any type of exercise and were asked to revert to their level of activity at baseline and maintain their current lifestyle or the RCTs do not mention whether people in control group do some exercise.

Reference

1. O'Brien K, Nixon S, Tynan AM, et al. Aerobic exercise interventions for adults living with HIV/AIDS. Cochrane Database Syst Rev. 2010;(8):CD001796

2. Busch AJ, Webber SC, Richards RS, et al. Resistance exercise training for fibromyalgia. Cochrane Database Syst Rev. 2013 Dec 20;(12):CD010884

Appendix 3 Results of Results of risk of bias

Study	Adequate sequence generation	Adequate allocation concealment	Blinding	Incomplete outcome data addressed	Free of selective reporting	Other bias
Kwon 2010	low risk	unclear risk	low risk	low risk	low risk	unclear risk
Okada 2010	low risk	unclear risk	unclear risk	low risk	low risk	unclear risk
KU 2010	low risk	unclear risk	unclear risk	unclear risk	unclear risk	unclear risk
Aylin 2009	low risk	unclear risk	unclear risk	low risk	low risk	low risk
Kenneth 2013	low risk	low risk	low risk	high risk	low risk	unclear risk
Larose 2011	low risk	unclear risk	unclear risk	low risk	low risk	low risk
Stolinskia 2008	low risk	unclear risk	unclear risk	low risk	low risk	unclear risk
Arslan 2014	high risk	high risk	unclear risk	low risk	unclear risk	high risk
Shenoy 2010	low risk	low risk	unclear risk	low risk	low risk	unclear risk
Belli 2011	low risk	low risk	low risk	low risk	low risk	low risk
Dunstan 1998	low risk	low risk	unclear risk	low risk	unclear risk	unclear risk
Church 2011	low risk	low risk	low risk	low risk	unclear risk	low risk
Kwon. 2010	low risk	unclear risk	unclear risk	unclear risk	low risk	unclear risk
Reid 2010	low risk	low risk	unclear risk	unclear risk	low risk	unclear risk
Sigal 2007	low risk	low risk	low risk	low risk	low risk	low risk
Choi 2012	low risk	low risk	low risk	low risk	unclear risk	low risk
Arora 2007	low risk	unclear risk	unclear risk	low risk	unclear risk	unclear risk
YAVARI 2010	low risk	unclear risk	unclear risk	low risk	unclear risk	low risk
Oliveira 2012	low risk	unclear risk	unclear risk	low risk	low risk	unclear risk
JENNINGS 2009	high risk	high risk	unclear risk	high risk	unclear risk	low risk
Cauza 2005	low risk	unclear risk	unclear risk	low risk	low risk	low risk
Cheung 2009	low risk	low risk	unclear risk	unclear risk	unclear risk	low risk
Franciele 2013	low risk	low risk	unclear risk	low risk	unclear risk	low risk
Whye 2011	low risk	unclear risk	low risk	low risk	low risk	low risk

Morton 2012	low risk	high risk	unclear risk	low risk	low risk	low risk
Dede 2014	low risk	high risk	unclear risk	low risk	low risk	low risk
BACCHI 2012	low risk	low risk	low risk	low risk	unclear risk	low risk
Ng 2010	low risk	low risk	unclear risk	high risk	low risk	low risk
Sparks 2013	low risk	unclear risk	unclear risk	low risk	low risk	unclear risk
Gavin 2010	low risk	low risk	unclear risk	low risk	low risk	low risk
Winnick 2008	low risk	unclear risk	unclear risk	low risk	low risk	low risk
MADDEN 2009	low risk	low risk	low risk	low risk	low risk	unclear risk
Madden 2011	low risk	low risk	low risk	low risk	unclear risk	unclear risk
William 2011	low risk	high risk	unclear risk	low risk	low risk	low risk
Kadoglou 2014	low risk	high risk	unclear risk	low risk	low risk	unclear risk
ALAM 2004	low risk	unclear risk	unclear risk	low risk	low risk	unclear risk
Tessier 2000	low risk	low risk	unclear risk	low risk	low risk	low risk

Appendix 4 Results of direct, indirect, and network meta-analyses

Appendix 4-1 Results of network meta-analyses on HbA1c

Comparisons	Direct evidence				Indirect evidence	Network meta-analysis	Inconsistency
	Number of RCTs	Heterogeneity (P-value)	RoM [95%CI]	Contribute to NMA/%	RoM [95%CI]	RoM [95%CI]	p-value
No exercise (reference)							
Supervised aerobic	11	0.0009	**0.96 [0.94; 0.98]**	55	**0.95 [0.93; 0.97]**	**0.96 [0.94; 0.97]**	0.4969
Supervised resistance	5	0.009	**0.96 [0.94; 0.98]**	46	**0.96 [0.94; 0.98]**	**0.96 [0.95; 0.97]**	0.8366
Unsupervised aerobic	2	0.1243	0.98 [0.90; 1.06]	53	1.05 [1.00; 1.10]	1.03 [1.00; 1.07]	0.0044
Unsupervised resistance	2	0.0017	0.98 [0.90; 1.06]	26	1.05 [1,00; 1.10]	1.03 [0.99; 1.07]	0.1605
Combined (reference)							
Flexibility	1	0.0009	1.16 [0.88; 1.53]	31	1.14 [0.95; 1.38]	1.15 [0.98; 1.34]	0.7441
No exercise	3	0.0009	**1.07 [1.04; 1.09]**	47	**1.08 [1.06; 1.11]**	**1.08 [1.06; 1.09]**	0.4204
Supervised aerobic	4	0.0009	**1.03 [1.01; 1.04]**	45	**1.03 [1.01; 1.06]**	**1.03 [1.02; 1.05]**	0.4248
Supervised resistance	4	0.0009	**1.03 [1.01; 1.06]**	47	**1.03 [1.01; 1.06]**	**1.03 [1.02; 1.05]**	0.9388
Unsupervised aerobic	0		-	0	**1.11 [1.07; 1.15]**	**1.11 [1.07; 1.15]**	-
Unsupervised resistance	0		-	0	**1.11 [1.06; 1.16]**	**1.11 [1.06; 1.16]**	-
Flexibility (reference)							

No exercise	0		-	0	0.94 [0.80; 1.09]	0.94 [0.80; 1.09]	-
Supervised aerobic	1	0.0009	0.87 [0.66; 1.14]	32	0.91 [0.75; 1.10]	0.89 [0.76; 1.05]	0.455
Supervised resistance	1	0.0009	0.93 [0.72; 1.20]	23	0.88 [0.72; 1.07]	0.90 [0.77; 1.05]	0.6491
Unsupervised aerobic	0		-	0	0.97 [0.83; 1.14]	0.97 [0.83; 1.14]	-
Unsupervised resistance	0		-	0	0.96 [0.82; 1.13]	0.96 [0.82; 1.13]	-
Supervised aerobic (reference)							
Supervised resistance	8	0.009	1.00 [0.98; 1.02]	53	1.01 [0.99; 1.03]	1.01 [0.99; 1.02]	0.7070
Unsupervised aerobic	2	0.9954	**1.19 [1.13; 1.27]**	32	1.00 [0.96; 1.04]	**1.08 [1.05; 1.12]**	0.0001
Unsupervised resistance	0		-	0	**1.08 [1.03; 1.12]**	**1.08 [1.03; 1.12]**	-
Supervised resistance (reference)							
Unsupervised aerobic	1	0.0009	1.07 [0.95; 1.21]	8	**1.05 [1.01; 1.09]**	**1.08 [1.04; 1.12]**	0.9086
Unsupervised resistance	0		--	0	**1.07 [1.03; 1.12]**	**1.07 [1.03; 1.12]**	-
Unsupervised aerobic (reference)							
Unsupervised resistance	3	0.0017	1.00 [0.97; 1.04]	100	0.97 [0.88; 1.05]	1.00 [0.97; 1.03]	0.1606

NOTE: Comparison: Treatment comparison; p-value: p-value of test for disagreement (direct versus indirect); Bold: significant difference in direct analysis indirect analysis and network meta-analysis, RoM: ratio of mean; NMA: network meta-analysis.

Appendix 4-2 Results of network meta-analyses on FBG

Comparisons	Direct evidence				Indirect evidence	Network meta-analysis	Inconsistency
	Number of RCTs	Heterogeneity (P-value)	RoM [95%CI]	Contribute to NMA/%	RoM [95%CI]	RoM [95%CI]	p-value
**No exercise (reference)**							
Supervised aerobic	5	0.1597	**0.93 [0.87; 0.98]**	92	0.94 [0.77; 1.14]	**0.93 [0.88; 0.98]**	0.9014
Supervised resistance	2	0.6279	1.02 [0.87; 1.21]	37	1.01 [0.89; 1.14]	1.01 [0.92; 1.12]	0.9013
Unsupervised aerobic	0				1.11 [0.81; 1.52]	1.11 [0.81; 1.52]	-
Unsupervised resistance	0		-	0	1.03 [0.72; 1.47]	1.03 [0.72; 1.47]	-
**Anaerobic (reference)**							
Combined	0		-	0	0.98 [0.84; 1.15]	0.98 [0.84; 1.15]	-
No exercise	0		-	0	1.05 [0.97; 1.14]	1.05 [0.97; 1.14]	-
Supervised aerobic	2	0.1935	0.98 [0.92; 1.03]	100	-	0.98 [0.92; 1.03]	-
Supervised resistance	0	0	-	0	1.07 [0.96; 1.19]	1.07 [0.96; 1.19]	-
Unsupervised aerobic	0	0	-	0	1.17 [0.86; 1.60]	1.17 [0.86; 1.60]	-
Unsupervised resistance	0	0	-	0	1.08 [0.76; 1.54]	1.08 [0.76; 1.54]	-
**Combined (reference)**							
No exercise	1	1	1.07 [0.94; 1.22]	100	-	1.07 [0.94; 1.22]	

Supervised aerobic	0		-		0.99 [0.86; 1.15]	0.99 [0.86; 1.15]	-
Supervised resistance	0		-	-	1.09 [0.92; 1.28]	1.09 [0.92; 1.28]	-
Unsupervised aerobic	0		-	-	1.19 [0.85; 1.68]	1.19 [0.85; 1.68]	-
Unsupervised resistance	0		-	0	1.10 [0.76; 1.61]	1.10 [0.76; 1.61]	-
**Supervised aerobic (reference)**							
Supervised resistance	3	0.9287	1.09 [0.98; 1.22]	71	1.10 [0.93; 1.31]	1.09 [1.00; 1.20]	0.9014
Unsupervised aerobic	1	1	1.20 [0.88; 1.63]	-	-	1.20 [0.88; 1.63]	-
Unsupervised resistance	0		-	0	1.11 [0.78; 1.57]	1.11 [0.78; 1.57]	-
**Supervised resistance (reference)**							
Unsupervised aerobic	0		-		1.10 [0.80; 1.51]	1.10 [0.80; 1.51]	-
Unsupervised resistance	0	0	-	0	1.01 [0.71; 1.45]	1.01 [0.71; 1.45]	-
**Unsupervised aerobic (reference)**							
Unsupervised resistance	1	1	0.92 [0.79; 1.09]	100		0.92 [0.79; 1.09]	-

NOTE: Comparison: Treatment comparison; p-value: p-value of test for disagreement (direct versus indirect); FBG: fasting plasma glucose; Bold: significant difference in direct analysis indirect analysis and network meta-analysis, RoM: ratio of mean; NMA: network meta-analysis.

Appendix 4-3 Results of network meta-analyses weight

Comparisons	Direct evidence				Indirect evidence	Network meta-analysis	Inconsistency
	Number of RCTs	Heterogeneity (P-value)	RoM [95%CI]	Contribute to NMA/%	RoM [95%CI]	RoM [95%CI]	p-value
**No exercise (reference)**							
Supervised aerobic	6	0.7864	0.98 [0.94; 1.01]	63	0.96 [0.91; 1.00]	0.97 [0.94; 1.00]	0.2230
Supervised resistance	3	0.8421	0.98 [0.93; 1.03]	51	0.97 [0.92; 1.02]	0.98 [0.94; 1.01]	0.9521
Unsupervised aerobic	1	1	0.99 [0.92; 1.07]	32	**1.07 [1.01; 1.13]**	1.04 [1.00; 1.08]	0.1073
Unsupervised resistance	1	1	0.96 [0.90; 1.03]	76	0.98 [0.87; 1.10]	0.97 [0.91; 1.02]	0.8585
**Anaerobic (reference)**							
Combined	0		-	0	1.00 [0.91; 1.11]	1.00 [0.91; 1.11]	-
No exercise	0		-	0	1.07 [0.98; 1.17]	1.07 [0.98; 1.17]	-
Supervised aerobic	3	0.7726	1.05 [0.97; 1.14]	100	-	1.05 [0.97; 1.14]	**-**
Supervised resistance	0	0	-	0	1.05 [0.96; 1.15]	1.05 [0.96; 1.15]	-
Unsupervised aerobic	0	0	-	0	**1.12 [1.02; 1.22]**	**1.12 [1.02; 1.22]**	-
Unsupervised resistance	0	0	-	0	1.04 [0.93; 1.15]	1.04 [0.93; 1.15]	-
**Combined (reference)**							
No exercise	2	0.5385	1.07 [0.99; 1.15]	56	1.07[0.98; 1.16]	**1.07 [1.01; 1.13]**	0.9564

Supervised aerobic	1	1	1.05 [0.94; 1.18]	25	1.05 [0.98; 1.12]	1.05 [0.99; 1.11]	0.9433
Supervised resistance	1	1	1.05 [0.99; 1.11]	31	1.05 [0.98; 1.12]	1.05 [0.99; 1.11]	0.9951
Unsupervised aerobic	0		-	-	**1.11 [1.04; 1.19]**	**1.11 [1.04; 1.19]**	-
Unsupervised resistance	0		-	0	1.03 [0.95; 1.12]	1.03 [0.95; 1.12]	-
**Supervised aerobic (reference)**							
Supervised resistance	4	0.9547	1.01 [0.96; 1.05]	61	1.01 [0.96; 1.07]	1.01 [0.97; 1.05]	0.9495
Unsupervised aerobic	1	1	**1.09 [1.04; 1.14]**	69	1.02 [0.95; 1.10]	**1.07 [1.03; 1.11]**	0.0845
Unsupervised resistance	0		-	0	1.00 [0.94; 1.06]	1.00 [0.94; 1.06]	-
**Supervised resistance (reference)**							
Unsupervised aerobic	0		-	0	**1.06 [1.01; 1.11]**	**1.06 [1.01; 1.11]**	-
Unsupervised resistance	0	0	-	0	0.99 [0.92; 1.06]	0.99 [0.92; 1.06]	-
**Unsupervised aerobic (reference)**							
Unsupervised resistance	1	1	0.94 [0.84; 1.05]	35	0.93 [0.86; 1.01]	**0.93 [0.87; 0.99]**	0.7478

NOTE: Comparison: Treatment comparison; p-value: p-value of test for disagreement (direct versus indirect); Bold: significant difference in direct analysis indirect analysis and network meta-analysis, RoM: ratio of mean; NMA: network meta-analysis.

Appendix 4-4 Results of network meta-analyses on SBP

Comparisons	Direct evidence				Indirect evidence	Network meta-analysis	Inconsistency
	Number of RCTs	Heterogeneity (P-value)	RoM [95%CI]	Contribute to NMA/%	RoM [95%CI]	RoM [95%CI]	p-value
**No exercise (reference)**							
Supervised aerobic	6	0.2179	0.97 [0.94 1.00]	74	0.97 [0.92 1.02]	0.97 [0.95 1.00]	0.9455
Supervised resistance	3	0.0075	0.96 [0.92; 1.00]	51	0.96 [0.92; 1.00]	**0.96 [0.93; 0.99]**	0.8645
Unsupervised aerobic	2	0.2631	**0.94 [0.89; 0.99]**	66	1.03 [0.96; 1.11]	0.97 [0.93; 1.01]	0.0373
Unsupervised resistance	0				0.95 [0.87; 1.04]	0.95 [0.87; 1.04]	0.8585
**Anaerobic (reference)**							
Combined	0		-	0	0.98 [0.89; 1.08]	0.98 [0.89; 1.08]	-
No exercise	0		-	0	1.01 [0.92; 1.11]	1.01 [0.92; 1.11]	-
Supervised aerobic	2	0.8607	0.99 [0.90; 1.07]	100	-	0.99 [0.90; 1.07]	-
Supervised resistance	0	0	-	0	0.97 [0.89; 1.07]	0.97 [0.89; 1.07]	-
Unsupervised aerobic	0	0	-	0	0.98 [0.89; 1.08]	0.98 [0.89; 1.08]	-
Unsupervised resistance	0	0	-	0	0.96 [0.85; 1.09]	0.96 [0.85; 1.09]	-
**Combined (reference)**							
No exercise	2	0.9884	1.00 [0.94; 1.06]	46	1.07 [1.01; 1.13]	1.03 [0.99; 1.08]	0.1210

Supervised aerobic	1	1	1.02 [0.94; 1.10]	31	1.00 [0.95; 1.06]	1.01 [0.97; 1.05]	0.8008
Supervised resistance	1	1	1.02 [0.94; 1.10]	32	0.98 [0.93; 1.04]	0.99 [0.95; 1.04]	0.5126
Unsupervised aerobic	1	1	1.08 [0.95; 1.23]	18	0.98 [0.93; 1.04]	1.00 [0.95; 1.06]	0.1817
Unsupervised resistance	0			0	0.98 [0.89; 1.09]	0.98 [0.89; 1.09]	-
**Supervised aerobic (reference)**							
Supervised resistance	4	0.8527	0.99 [0.95; 1.04]	49	0.98 [0.94; 1.03]	0.99 [0.96; 1.02]	0.8030
Unsupervised aerobic	0				0.99 [0.95; 1.04]	0.99 [0.95; 1.04]	0.0845
Unsupervised resistance	0		-	0	0.98 [0.89; 1.07]	0.98 [0.89; 1.07]	-
**Supervised resistance (reference)**							
Unsupervised aerobic	1	1	1.05 [0.97; 1.14]	34	0.98 [0.93; 1.04]	1.01 [0.96; 1.05]	0.1797
Unsupervised resistance	0	0	-	0	0.99 [0.90; 1.09]	0.99 [0.90; 1.09]	-
**Unsupervised aerobic (reference)**							
Unsupervised resistance	1	1	0.98 [0.91; 1.07]	100	-	0.98 [0.91; 1.07]	0.7478

NOTE: Comparison: Treatment comparison; p-value: p-value of test for disagreement (direct versus indirect); SBP: systolic blood pressure; Bold: significant difference in direct analysis indirect analysis and network meta-analysis, RoM: ratio of mean; NMA: network meta-analysis.

Appendix 4-5 Results of network meta-analyses on DBP

Comparisons	Direct evidence				Indirect evidence	Network meta-analysis	Inconsistency
	Number of RCTs	Heterogeneity (P-value)	RoM [95%CI]	Contribute to NMA/%	RoM [95%CI]	RoM [95%CI]	p-value
**No exercise (reference)**							
Supervised aerobic	6	0.0103	0.97 [0.93; 1.01]	79	0.96 [0.91; 1.03]	0.97 [0.94; 1.00]	0.8631
Supervised resistance	3	0.0483	0.97 [0.92; 1.03]	55	0.96 [0.91; 1.01]	0.97 [0.93; 1.00]	0.7194
Unsupervised aerobic	2	0.0086	**0.92 [0.86; 0.98]**	80	1.03 [0.92; 1.15]	0.94 [0.89; 1.00]	0.0733
Unsupervised resistance	0				0.97 [0.86; 1.09]	0.97 [0.86; 1.09]	-
**Anaerobic (reference)**							
Combined	0		-	0	0.95 [0.86; 1.04]	0.95 [0.86; 1.04]	-
No exercise	0		-	0	0.96 [0.88; 1.04]	0.96 [0.88; 1.04]	-
Supervised aerobic	2	0.5039	0.93 [0.86; 1.00]	100		0.93 [0.86; 1.00]	**-**
Supervised resistance	0	0	-	0	0.93 [0.85; 1.01]	0.93 [0.85; 1.01]	-
Unsupervised aerobic	0	0	-	0	0.91 [0.82; 1.00]	0.91 [0.82; 1.00]	-
Unsupervised resistance	0	0	-	0	0.93 [0.81; 1.07]	0.93 [0.81; 1.07]	-
**Combined (reference)**							
No exercise	2	0.6432	0.99 [0.92; 1.07]	49	1.03 [0.96; 1.11]	1.01 [0.96; 1.06]	0.4556

Supervised aerobic	1	1	0.99 [0.90; 1.09]	33	0.97 [0.91; 1.04]	0.98 [0.93; 1.03]	0.8240
Supervised resistance	1	1	1.00 [0.91; 1.10]	35	0.96 [0.90; 1.03]	0.98 [0.92; 1.03]	0.5428
Unsupervised aerobic	0				0.95 [0.89; 1.03]	0.95 [0.89; 1.03]	
Unsupervised resistance	0			0	0.98 [0.86; 1.11]	0.98 [0.86; 1.11]	-
**Supervised aerobic (reference)**							
Supervised resistance	4	0.8270	1.00 [0.95; 1.06]	41	0.99 [0.94; 1.05]	1.00 [0.96; 1.04]	0. 0.7358
Unsupervised aerobic	0				0.97 [0.92; 1.04]	0.97 [0.92; 1.04]	
Unsupervised resistance	0		-	0	1.00 [0.89; 1.13]	0.99 [0.91; 1.08]	-
**Supervised resistance (reference)**							
Unsupervised aerobic	1	1	1.05 [0.95; 1.16]	28	0.94 [0.87; 1.01]	0.98 [0.92; 1.04]	0.0733
Unsupervised resistance	0	0	-	0	1.00 [0.89; 1.13]	1.00 [0.89; 1.13]	-
**Unsupervised aerobic (reference)**							
Unsupervised resistance	1	1	1.03 [0.95; 1.11]	100	-	1.03 [0.93; 1.14]	-

NOTE: Comparison: Treatment comparison; p-value: p-value of test for disagreement (direct versus indirect); DBP: diastolic blood pressure; Bold: significant difference in direct analysis indirect analysis and network meta-analysis, RoM: ratio of mean; NMA: network meta-analysis.

Appendix 4-6 Results of network meta-analyses on TC

Comparisons	Direct evidence				Indirect evidence	Network meta-analysis	Inconsistency
	Number of RCTs	Heterogeneity (P-value)	RoM [95%CI]	Contribute to NMA/%	RoM [95%CI]	RoM [95%CI]	p-value
**No exercise (reference)**							
Supervised aerobic	4		**0.89 [0.83; 0.95]**	66	**0.91 [0.83; 0.99]**	**0.89 [0.85; 0.94]**	0.7329
Supervised resistance	1		**0.86 [0.77; 0.95]**	32	**0.90 [0.83; 0.96]**	**0.88 [0.83; 0.94]**	0.5024
Unsupervised aerobic	2		0.97 [0.89; 1.04]	68	0.95 [0.85; 1.06]	0.96 [0.90; 1.02]	0.7674
**Combined (reference)**							
Flexibility	1		0.94 [0.80; 1.10]	40	0.91 [0.80; 1.04]	0.92 [0.84; 1.02]	0.7557
No exercise	2		0.96 [0.90; 1.03]	74	1.01 [0.91; 1.13]	0.97 [0.92; 1.03]	0.4225
Supervised aerobic	1		0.93 [0.79; 1.08]	18	**0.86; [0.80; 0.93]**	**0.87 [0.82; 0.93]**	0.3910
Supervised resistance	11		0.86 [0.74; 1.00]	22	**0.86 [0.79; 0.93]**	**0.86 [0.80; 0.92]**	0.9888
Unsupervised aerobic	0		-	0	0.93 [0.86; 1.01]	0.93 [0.86; 1.01]	-
**Flexibility (reference)**							
No exercise	0		-	0	1.05 [0.95; 1.17]	1.05 [0.95; 1.17]	-
Supervised aerobic	1		0.94 [0.85; 1.04]	37	0.98 [0.84; 1.16]	0.92 [0.81; 1.04]	0.5207
Supervised resistance	1		0.93 [0.84; 1.03]	40	0.91 [0.78; 1.07]	0.94 [0.83; 1.07]	0.7433

Unsupervised aerobic	0	-	0	1.01 [0.90; 1.13]	1.01 [0.90; 1.13]	-
**Supervised aerobic (reference)**						
Supervised resistance	3	0.99 [0.91; 1.08]	47	0.98 [0.90; 1.07]	0.99 [0.93; 1.05]	0.8675
Unsupervised aerobic	2	1.20 [0.93; 1.55]	8	1.06 [0.98; 1.15]	1.07 [1.00; 1.15]	0.3503
**Supervised resistance (reference)**						
Unsupervised aerobic	1	1.06 [0.96; 1.16]	49	**1.12 [1.01; 1.23]**	**1.09 [1.01; 1.16]**	0.4284

NOTE: Comparison: Treatment comparison; p-value: p-value of test for disagreement (direct versus indirect); TC: total cholesterol; Bold: significant difference in direct analysis indirect analysis and network meta-analysis, RoM: ratio of mean; NMA: network meta-analysis.

Appendix 4-7 Results of network meta-analyses on TG

Comparisons	Direct evidence				Indirect evidence	Network meta-analysis	Inconsistency
	Number of RCTs	Heterogeneity (P-value)	RoM [95%CI]	Contribute to NMA/%	RoM [95%CI]	RoM [95%CI]	p-value
**No exercise (reference)**							
Supervised aerobic	6	0.9480	**0.88 [0.79; 0.98]**	72	0.86 [0.73; 1.02]	**0.87 [0.80; 0.96]**	0.8720
Supervised resistance	3	0.9460	**0.85 [0.75; 0.97]**	48	0.92 [0.81; 1.04]	**0.89 [0.81; 0.97]**	0.4518
Unsupervised aerobic	2	0.3147	0.95 [0.84; 1.08]	62	0.95 [0.81; 1.12]	0.95 [0.86; 1.05]	0.9996
**Combined (reference)**							
No exercise	4	0.6096	**1.28 [1.10; 1.50]**	56	**1.41 [1.18; 1.68]**	**1.34 [1.19; 1.50]**	0.4360
Supervised aerobic	2	0.8472	1.23 [0.98; 1.53]	34	1.14 [0.97; 1.33]	**1.17 [1.03; 1.33]**	0.5952
Supervised resistance	2	0.8558	1.23 [0.98; 1.53]	34	1.16 [0.99; 1.36]	**1.18 [1.04; 1.35]**	0.7048
Unsupervised aerobic	0				**1.27 [1.10; 1.47]**	**1.27 [1.10; 1.47]**	
**Supervised aerobic (reference)**							
Supervised resistance	4	0.3315	1.07 [0.87; 1.30]	31	0.99 [0.87; 1.14]	1.01 [0.91; 1.14]	0.5631
Unsupervised aerobic	1	1	1.90 [0.34; 10.48]	8	1.09 [0.95; 1.24]	1.09 [0.96; 1.24]	0.5231
**Supervised resistance (reference)**							

Unsupervised aerobic	1	1	1.07 [0.94; 1.22]	62	1.08 [0.91; 1.28]	1.08 [0.97; 1.19]	0.9377

NOTE: Comparison: Treatment comparison; p-value: p-value of test for disagreement (direct versus indirect); TG: triacylglycerol; Bold: significant difference in direct analysis indirect analysis and network meta-analysis, RoM: ratio of mean; NMA: network meta-analysis.

Appendix 4-8 Results of network meta-analyses on LDL

Comparisons	Direct evidence				Indirect evidence	Network meta-analysis	Inconsistency
	Number of RCTs	Heterogeneity (P-value)	RoM [95%CI]	Contribute to NMA/%	RoM [95%CI]	RoM [95%CI]	p-value
**No exercise (reference)**							
Supervised aerobic	5	0.1477	0.90 [0.79; 1.02]	68	0.87 [0.73; 1.04]	**0.89 [0.80; 0.99]**	0.7115
Supervised resistance	1	0.6279	1.04 [0.77; 1.40]	24	**0.83 [0.71; 0.98]**	0.88 [0.76; 1.01]	0.2127
Unsupervised aerobic	1	0.5010	0.98 [0.73; 1.32]	48	1.17 [0.89; 1.55]	1.08 [0.88; 1.33]	0.3952
Unsupervised resistance	0				1.12 [0.81; 1.56]	1.12 [0.81; 1.56]	
**Combined (reference)**							
Flexibility	1	0.1786	0.89 [0.64; 1.24]	41	1.01 [0.77; 1.33]	0.96 [0.78; 1.19]	0.5713
No exercise	3	0.2117	1.13 [0.95; 1.34]	54	1.03 [0.85; 1.24]	1.08 [0.95; 1.23]	0.4633
Supervised aerobic	2	0.1312	0.97 [0.77; 1.21]	34	0.96 [0.82; 1.13]	0.96 [0.85; 1.10]	0.9891
Supervised resistance	2	0.1751	0.91 [0.73; 1.14]	43	0.98 [0.81; 1.18]	0.95 [0.82; 1.10]	0.6454
Unsupervised aerobic	0		-	0	1.17 [0.93; 1.47]	1.17 [0.93; 1.47]	-
Unsupervised resistance	0		-	0	1.22 [0.86; 1.72]	1.22 [0.86; 1.72]	-
**Flexibility (reference)**							
No exercise	0		-	0	1.13 [0.91; 1.41]	1.13 [0.91; 1.41]	-

Supervised aerobic	1	0.1333	1.03 [0.72; 1.46]	36	0.99 [0.76; 1.29]	1.00 [0.81; 1.24]	0.8750
Supervised resistance	1	0.2179	0.89 [0.64; 1.26]	38	1.05 [0.80; 1.38]	0.99 [0.80; 1.22]	0.4626
Unsupervised aerobic	0		-	0	1.22 [0.92; 1.62]	1.21 [0.94; 1.57]	-
Unsupervised resistance	0		-	0	1.27 [0.86; 1.86]	1.27 [0.86; 1.86]	-
**Supervised aerobic (reference)**							
Supervised resistance	4	0.3452	0.98 [0.82; 1.17]	54	0.99 [0.82; 1.21]	0.99 [0.86; 1.12]	0.9229
Unsupervised aerobic	2	0.1327	**1.30 [1.01; 1.69]**	59	1.09 [0.79; 1.50]	1.21 [0.99; 1.48]	0.3952
Unsupervised resistance	0		-	0	1.26 [0.91; 1.75]	1.26 [0.91; 1.75]	-
**Supervised resistance (reference)**							
Unsupervised aerobic	0				1.23 [0.98; 1.56]	1.23 [0.98; 1.56]	
Unsupervised resistance	0		--	0	1.28 [0.90; 1.81]	1.28 [0.90; 1.81]	-
**Unsupervised aerobic (reference)**							
Unsupervised resistance	1	0.3452	1.04 [0.80; 1.35]	100	-	1.04 [0.80; 1.35]	

NOTE: Comparison: Treatment comparison; p-value: p-value of test for disagreement (direct versus indirect); LDL: low-density lipoprotein cholesterol; Bold: significant difference in direct analysis indirect analysis and network meta-analysis, RoM: ratio of mean; NMA: network meta-analysis.

Appendix 4-9 Results of network meta-analyses on HDL

Comparisons	Direct evidence				Indirect evidence	Network meta-analysis	Inconsistency
	Number of RCTs	Heterogeneity (P-value)	RoM [95%CI]	Contribute to NMA/%	RoM [95%CI]	RoM [95%CI]	p-value
**No exercise (reference)**							
Supervised aerobic	5	0.0003	0.92 [0.89; 0.96]	68	0.90 [0.86; 0.95]	0.92 [0.89; 0.96]	0.1271
Supervised resistance	2	0.8616	1.04 [0.94; 1.16]	23	0.86 [0.81; 0.91]	0.90 [0.85; 0.94]	0.0014
Unsupervised aerobic	2	0.8822	1.03 [0.88; 1.20]	70	0.90 [0.71; 1.13]	0.99 [0.87; 1.12]	0.3290
Unsupervised resistance	0				0.91 [0.71; 1.17]	0.91 [0.71; 1.17]	
**Combined (reference)**							
Flexibility	1	0.1786	0.92 [0.79; 1.06]	33	1.00 [0.90; 1.10]	0.97 [0.89; 1.05]	0.3502
No exercise	3	0.2117	1.04 [1.00; 1.08]	79	0.98[0.91; 1.06]	1.03 [1.00; 1.07]	0.1381
Supervised aerobic	2	0.1312	0.96 [0.87; 1.05]	23	0.95 [0.90; 1.00]	**0.95 [0.91; 0.99]**	0.8302
Supervised resistance	2	0.1751	0.88 [0.81; 0.96]	37	0.95 [0.89; 1.02]	**0.92 [0.88; 0.97]**	0.1245
Unsupervised aerobic	0		-	0	1.01 [0.93; 1.11]	1.01 [0.93; 1.11]	-
Unsupervised resistance	0		-	0	0.93 [0.79; 1.11]	0.93 [0.79; 1.11]	-
**Flexibility (reference)**							
No exercise	0		-	0	1.06 [0.98; 1.16]	1.06 [0.98; 1.16]	-

Supervised aerobic	1	0.1333	0.95 [0.86; 1.06]	32	0.99 [0.76; 1.29]	0.98 [0.90; 1.06]	0.3705
Supervised resistance	1	0.2179	0.89 [0.79; 1.00]	48	1.02 [0.91; 1.14]	0.95 [0.88; 1.03]	0.0900
Unsupervised aerobic	0		-	0	1.04 [0.93; 1.17]	1.04 [0.93; 1.17]	-
Unsupervised resistance	0		-	0	0.96 [0.80; 1.16]	0.96 [0.80; 1.16]	-
**Supervised aerobic (reference)**							
Supervised resistance	4	0.1382	0.96 [0.86; 1.08]	51	0.99 [0.88; 1.11]	0.98 [0.90; 1.06]	0.7298
Unsupervised aerobic	1	1	0.74 [0.35; 1.57]	3	1.03 [0.90; 1.19]	1.02 [0.89; 1.17]	0.3930
Unsupervised resistance	0		-	0	0.94 [0.73; 1.22]	1.26 [0.95; 1.67]	-
**Supervised resistance (reference)**							
Unsupervised aerobic	1	1	0.98 [0.78; 1.23]	37	1.08 [0.91; 1.29]	1.05 [0.91; 1.20]	0.4963
Unsupervised resistance	0		--	0	0.96 [0.75; 1.25]	0.96 [0.75; 1.25]	-
**Unsupervised aerobic (reference)**							
Unsupervised resistance	1		0.92 [0.74; 1.14]	100		0.92 [0.74; 1.14]	

NOTE: Comparison: Treatment comparison; p-value: p-value of test for disagreement (direct versus indirect); HDL: high-density lipoprotein cholesterol; Bold: significant difference in direct analysis indirect analysis and network meta-analysis, RoM: ratio of mean; NMA: network meta-analysis

Appendix 5 Subgroup analyses

Appendix 5-1 Subgroup analysis of diabetes duration in HbA1c

Comparisons	diabetes duration ≤6 (years)			diabetes duration ＞6 (years)		
	No. of direct comparisons	prop	NMA 95%-CI	No. of direct comparisons	prop	NMA 95%-CI
vs. Combined						
flexibility training	na	na	na	2	0.47	0.98 [0.92; 1.05]
no exercise	2	0.45	1.16 [1.12;1.21]	2	0.43	1.05 [1.05; 1.05]
supervised aerobic	2	0.44	1.07 [1.03;1.11]	3	0.58	1.03 [1.02; 1.03]
supervised resistance	2	0.46	1.07 [1.03;1.11]	3	0.54	1.02 [1.02; 1.03]
unsupervised aerobic	0	0	1.09 [1.00; 1.19]	na	na	na
unsupervised resistance	0	0	1.11 [1.01; 1.23]	na	na	na
vs. no exercise						
supervised aerobic	4	0.66	0.92 [0.89; 0.95]	5	0.44	0.98 [0.97; 0.98]
supervised resistance	3	0.51	0.92 [0.89; 0.95]	2	0.43	0.97 [0.97; 0.98]
unsupervised aerobic	1	0.54	0.94 [0.86; 1.02]	na	na	na
unsupervised resistance	1	1	0.96 [0.87; 1.05]	na	na	na
vs. supervised aerobic						
supervised resistance	3	0.44	1.01 [0.97; 1.04]	6	0.58	1.00 [0.99; 1.00]
unsupervised aerobic	0	0	1.02 [0.94; 1.12]	na	na	na
unsupervised resistance	0	0	1.04 [0.95; 1.15]	na	na	na
vs. supervised resistance						
unsupervised aerobic	1	0.5	1.02 [0.94; 1.11]	na	na	na
unsupervised resistance	0	0	1.04 [0.94; 1.15]	na	na	na
vs. unsupervised aerobic						
unsupervised resistance	0	0	1.02 [0.90; 1.16]	na	na	na
vs. flexibility training						
no exercise	na	na	na	0	0	1.07 [1.00; 1.14]
supervised aerobic	na	na	na	2	0.31	1.04 [0.98; 1.11]
supervised resistance	na	na	na	2	0.22	1.04 [0.97; 1.11]

NOTE: NA: not available; prop: direct evidence proportion; NMA: network meta-analysis

Appendix 5-2 Subgroup analysis of age in HbA1c

Comparisons	Age＜60 (years)			Age≥60 (years)		
	No. of direct comparisons	prop	NMA 95%-CI	No. of direct comparisons	prop	NMA 95%-CI
vs. Combined						
flexibility training	2	0.47	0.99 [0.92; 1.06]	na	na	na
no exercise	4	0.47	1.07 [1.06; 1.09]	na	na	na
supervised aerobic	5	0.45	1.03 [1.01; 1.04]	na	na	na
supervised resistance	5	0.47	1.03 [1.02; 1.05]	na	na	na
unsupervised aerobic	0	0	1.11 [1.07; 1.15]	na	na	na
unsupervised resistance	0	0	1.11 [1.06; 1.16]	na	na	na
vs. no exercise						
supervised aerobic	8	0.53	**0.96 [0.94; 0.97]**	3	1	0.94 [0.87; 1.02]
supervised resistance	5	0.46	0.96 [0.95; 0.98]	na	na	na
unsupervised aerobic	2	0.47	1.03 [1.00; 1.07]	na	na	na
unsupervised resistance	1	0.2	1.03 [0.99; 1.08]	1	1	1.03 [0.89; 1.19]
vs. supervised aerobic						
supervised resistance	9	0.54	1.01 [0.99; 1.02]	na	na	na
unsupervised aerobic	2	0.37	1.08 [1.05; 1.12]	na	na	na
unsupervised resistance	0	0	**1.08 [1.03; 1.13]**	0	0	1.09 [0.93; 1.29]
vs. supervised resistance						
unsupervised aerobic	1	0.09	1.08 [1.04; 1.12]	na	na	na
unsupervised resistance	0	0	1.07 [1.02; 1.12]	na	na	na
vs. unsupervised aerobic						
unsupervised resistance	3	0.92	1.00 [0.97; 1.03]	na	na	na
vs. flexibility training						
no exercise	0	0	1.09 [1.02; 1.17]	na	na	na
supervised aerobic	2	0.32	1.04 [0.97; 1.11]	na	na	na
supervised resistance	2	0.23	1.05 [0.98; 1.12]	na	na	na
unsupervised aerobic	0	0	1.13 [1.04; 1.21]	na	na	na
unsupervised resistance	0	0	1.12 [1.03; 1.21]	na	na	na

NOTE: NA: not available; prop: direct evidence proportion; NMA: network meta-analysis

Appendix 5-3 Subgroup analysis of study duration in HbA1c

Comparisons	Study duration ≤6 (month)			Study duration ＞6 (month)		
	No. of direct comparisons	prop	NMA 95%-CI	No. of direct comparisons	prop	NMA 95%-CI
vs. Combined						
flexibility training	na	na	na	1	0.32	1.18 [1.01; 1.39]
no exercise	1	0.47	1.06 [1.04; 1.07]	2	0.48	1.14 [1.10; 1.19]
supervised aerobic	1	0.46	1.02 [1.01; 1.04]	3	0.47	1.07 [1.03; 1.11]
supervised resistance	1	0.48	1.02 [1.01; 1.04]	3	0.49	1.08 [1.04; 1.12]
unsupervised aerobic	0	0	1.04 [1.00; 1.09]	0	0	1.28 [1.20; 1.37]
unsupervised resistance	0	0	1.04 [0.99; 1.09]	na	na	na
vs. no exercise						
supervised aerobic	8	0.57	0.97 [0.95; 0.98]	3	0.54	0.94 [0.90; 0.97]
supervised resistance	3	0.47	0.97 [0.95; 0.98]	2	0.5	0.94 [0.91; 0.98]
unsupervised aerobic	2	0.67	0.99 [0.95; 1.03]	0	0	**1.12 [1.05; 1.19]**
unsupervised resistance	2	0.31	0.98 [0.94; 1.03]	na	na	na
vs. supervised aerobic						
supervised resistance	5	0.54	1.00 [0.99; 1.01]	3	0.5	1.01 [0.97; 1.04]
unsupervised aerobic	0	0	1.02 [0.98; 1.06]	2	1	**1.19 [1.13; 1.26]**
unsupervised resistance	0	0	1.02 [0.97; 1.07]			
vs. supervised resistance						
unsupervised aerobic	1	0.12	1.02 [0.98; 1.06]	0	0	**1.19 [1.11; 1.27]**
unsupervised resistance	0	0	1.02 [0.97; 1.07]	na	na	na
vs. unsupervised aerobic						
unsupervised resistance	3	0.91	1.00 [0.97; 1.03]	na	na	na
vs. flexibility training						
no exercise	na	na	na	0	0	0.97 [0.83; 1.13]
supervised aerobic	na	na	na	1	0.32	0.91 [0.77; 1.06]
supervised resistance	na	na	na	1	0.38	0.91 [0.78; 1.07]

NOTE: NA: not available; prop: direct evidence proportion; NMA: network meta-analysis

Appendix 5-4 Subgroup analysis of type of patients in HbA1c

Comparisons	Sedentary			Non-sedentary		
	No. of direct comparisons	prop	NMA 95%-CI	No. of direct comparisons	prop	NMA 95%-CI
vs. Combined						
flexibility training	1	0.31	1.14 [0.98; 1.34]	NA	NA	NA
no exercise	2	0.43	1.05 [1.05; 1.06]	1	0.38	1.16 [1.03; 1.32]
supervised aerobic	3	0.58	**1.03 [1.02; 1.03]**	1	0.37	1.07 [0.94; 1.21]
supervised resistance	3	0.54	**1.02 [1.02; 1.03]**	1	0.39	1.07 [0.94; 1.22]
unsupervised aerobic	0	0	1.05 [1.00; 1.11]	0	0	1.17 [1.00; 1.36]
unsupervised resistance	0	0	1.08 [0.93; 1.25]	0	0	1.18 [0.99;1.41]
vs. no exercise						
supervised aerobic	4	0.44	0.98 [0.97; 0.98]	7	0.7	0.92 [0.86; 0.98]
supervised resistance	2	0.43	0.97 [0.97; 0.98]	3	0.46	0.92 [0.85; 1.00]
unsupervised aerobic	1	1	1.00 [0.95; 1.05]	1	0.24	1.00 [0.90; 1.11]
unsupervised resistance	1	1	1.03 [0.89; 1.19]	1	0.39	1.02 [0.89; 1.16]
vs. supervised aerobic						
supervised resistance	4	0.58	1.00 [0.99; 1.00]	4	0.53	1.01 [0.93; 1.09]
unsupervised aerobic	0	0	1.02 [0.97; 1.08]	2	0.48	1.09 [0.98; 1.21]
unsupervised resistance	0	0	1.05 [0.91; 1.22]	0	0	1.11 [0.97; 1.27]
vs. supervised resistance						
unsupervised aerobic	0	0	1.03 [0.98; 1.08]	1	0.27	1.09 [0.97; 1.22]
unsupervised resistance	0	0	1.06 [0.91; 1.22]	0	0	1.10 [0.95; 1.27]
vs. unsupervised aerobic						
unsupervised resistance	0	0	1.03 [0.88; 1.20]	3	0.80	1.02 [0.91; 1.13]
vs. flexibility training						
no exercise	0	0	0.92 [0.79; 1.07]	NA	NA	NA
supervised aerobic	1	0.31	0.90 [0.77; 1.05]	NA	NA	NA
supervised resistance	1	0.37	0.89 [0.77; 1.04]	NA	NA	NA

NOTE: NA: not available; prop: direct evidence proportion; NMA: network meta-analysis

Appendix 5-5 Subgroup analysis of diabetes duration in FBG

Comparisons	diabetes duration ≤6 (years)			diabetes duration ＞6 (years)		
	No. of direct comparisons	prop	NMA 95%-CI	No. of direct comparisons	prop	NMA 95%-CI
vs. Combined						
flexibility training	na	na	na	1	0.51	0.90 [0.79; 1.03]
no exercise	na	na	na	1	0.58	1.04 [0.94; 1.15]
supervised aerobic	na	na	na	1	0.21	0.98 [0.88; 1.08]
supervised resistance	na	na	na	1	0.2	1.11 [0.99; 1.26]
vs. no exercise						
supervised aerobic	2	0.9	**0.81 [0.68; 0.95]**	3	0.83	0.94 [0.88; 1.00]
supervised resistance	1	0.53	0.92 [0.69; 1.22]	1	0.26	1.07 [0.97; 1.18]
vs. supervised aerobic						
supervised resistance	1	0.56	1.14 [0.86; 1.51]	3	0.66	**1.14 [1.04; 1.25]**
vs. flexibility training						
no exercise	na	na	na	0	0	1.16 [1.01; 1.33]
supervised aerobic	na	na	na	1	0.44	1.09 [0.96; 1.23]
supervised resistance	na	na	na	1	0.32	1.24 [1.08; 1.42]

NOTE: NA: not available; prop: direct evidence proportion; NMA: network meta-analysis; FBG: fasting plasma glucose

Appendix 5-6 Subgroup analysis of age in FBG

Comparisons	Age＜60 (years)				Age≥60 (years)			
	No. of direct comparisons	prop	nma	95%-CI	No. of direct comparisons	prop	nma	95%-CI
vs. Combined								
flexibility training	1	0.5	0.88	[0.73; 1.05]	na	na	na	
no exercise	1	0.47	1.09	[0.93; 1.27]	na	na	na	
supervised aerobic	1	0.27	0.93	[0.80; 1.09]	na	na	na	
supervised resistance	1	0.25	1.09	[0.92; 1.28]	na	na	na	
unsupervised aerobic	0	0	1.12	[0.76; 1.65]	na	na	na	
unsupervised resistance	0	0	1.03	[0.65; 1.64]	na	na	na	
vs. no exercise								
supervised aerobic	3	0.73	0.86	[0.77; 0.95]	na	na	na	
supervised resistance	2	0.37	1.00	[0.88; 1.14]	na	na	na	
unsupervised aerobic	0	0	1.03	[0.71; 1.50]	na	na	na	
unsupervised resistance	0	0	0.95	[0.61; 1.49]	na	na	na	
vs. supervised aerobic								
supervised resistance	4	0.64	1.17	[1.04; 1.31]	na	na	na	
unsupervised aerobic	1	1	1.20	[0.84; 1.72]	na	na	na	
unsupervised resistance	0	0	1.11	[0.72; 1.71]	na	na	na	
vs. supervised resistance								
unsupervised aerobic	0	0	1.03	[0.71; 1.50]	na	na	na	
unsupervised resistance	0	0	0.95	[0.61; 1.49]	na	na	na	
vs. unsupervised aerobic								
unsupervised resistance	1	1	0.92	[0.72; 1.18]	na	na	na	
vs. flexibility training								
no exercise	0	0	1.24	[1.03; 1.50]			na	
supervised aerobic	1	0.43	1.06	[0.89; 1.27]	3	1	0.95	[0.82; 1.10]
supervised resistance	1	0.35	1.24	[1.04; 1.49]	na	na	na	
unsupervised aerobic	0	0	1.28	[0.86; 1.90]	na	na	na	
unsupervised resistance	0	0	1.18	[0.74; 1.89]	na	na	na	
vs. anaerobic								
no exercise	na	na	na		0	0	0.99	[0.80; 1.23]
supervised aerobic	na	na	na		2	1	0.94	[0.81; 1.10]

NOTE: NA: not available; prop: direct evidence proportion; NMA: network meta-analysis; FBG: fasting plasma glucose

Appendix 5-7 Subgroup analysis of study duration in FBG

Comparisons	Study duration ≤6 (month)			Study duration ＞6 (month)		
	No. of direct comparisons	prop	NMA 95%-CI	No. of direct comparisons	prop	NMA 95%-CI
vs. Combined						
no exercise	1	1	1.07 [0.94; 1.22]	na	na	na
supervised aerobic	0	0	1.02 [0.88; 1.19]	na	na	na
supervised resistance	0	0	1.11 [0.94; 1.31]	na	na	na
vs. no exercise						
supervised aerobic	4	0.87	0.95 [0.89; 1.02]	1	1	**0.88 [0.80; 0.97]**
supervised resistance	2	0.4	1.04 [0.93; 1.15]	na	na	na
unsupervised aerobic	na	na	na	0	0	1.06 [0.77; 1.46]
vs. supervised aerobic						
supervised resistance	3	0.73	1.08 [0.99; 1.19]	na	na	na
unsupervised aerobic	na	na	na	1	1	1.20 [0.88; 1.63]
vs. anaerobic						
combined	0	0	0.95 [0.81;1.12]	na	na	na
no exercise	0	0	1.02 [0.93;1.12]	na	na	na
supervised aerobic	2	1	0.98 [0.92;1.03]	na	na	na
supervised resistance	0	0	1.06 [0.95;1.18]	na	na	na

NOTE: NA: not available; prop: direct evidence proportion; NMA: network meta-analysis; FBG: fasting plasma glucose

Appendix 5-8 Subgroup analysis of type of patients in FBG

Comparisons	Sedentary			Non-sedentary		
	No. of direct comparisons	prop	NMA 95%-CI	No. of direct comparisons	prop	NMA 95%-CI
vs. Combined						
no exercise	na	na	na	1	1	1.07 [0.94; 1.22]
supervised aerobic	na	na	na	0	0	1.02 [0.88; 1.19]
supervised resistance	na	na	na	0	0	1.11 [0.94; 1.31]
unsupervised aerobic	na	na	na	0	0	1.23 [0.87; 1.73]
vs. no exercise						
supervised aerobic	1	1	**0.88 [0.80; 0.97]**	4	0.87	0.95 [0.89; 1.02]
supervised resistance	na	na	na	2	0.40	1.04 [0.93; 1.15]
unsupervised aerobic	na	na	na	0	0	1.15 [0.84; 1.57]
vs. supervised aerobic						
supervised resistance	na	na	na	3	0.73	1.08 [0.99; 1.19]
unsupervised aerobic	na	na	na	1	1	1.20 [0.88; 1.63]
vs. supervised resistance						
unsupervised aerobic	na	na	na	0	0	1.11 [0.80; 1.53]
vs. anaerobic						
combined				0	0	0.96 [0.82; 1.13]
no exercise	0	0	0.97 [0.78; 1.21]	0	0	1.03 [0.94; 1.13]
supervised aerobic	1	1	0.86 [0.70; 1.05]	1	1	0.99 [0.93; 1.05]
supervised resistance	na	na	na	0	0	1.07 [0.96; 1.19]
unsupervised aerobic	na	na	na	0	0	1.18 [0.86; 1.62]

NOTE: NA: not available; prop: direct evidence proportion; NMA: network meta-analysis; FBG: fasting plasma glucose

Appendix 5-9 Subgroup analysis of diabetes duration in weight

Comparisons	diabetes duration ≤6 (years)			diabetes duration ＞6 (years)		
	No. of direct comparisons	prop	NMA 95%-CI	No. of direct comparisons	prop	NMA 95%-CI
vs. Combined						
supervised aerobic	na	na	na	1	1	1.09 [0.98; 1.22]
supervised resistance	na	na	na	0	0	1.06 [0.95; 1.20]
unsupervised aerobic	na	na	na	0	0	1.07 [0.95; 1.20]
vs. no exercise						
supervised aerobic	1	0.94	1.02 [0.95; 1.08]	3	0.72	0.97 [0.93; 1.02]
supervised resistance	1	0.78	0.98 [0.88; 1.09]	1	0.54	0.98 [0.93; 1.02]
unsupervised resistance	1	1	0.96 [0.90; 1.03]	na	na	na
vs. supervised aerobic						
supervised resistance	1	0.27	0.96 [0.85; 1.09]	2	0.74	1.00 [0.96; 1.05]
unsupervised resistance	0	0	0.95 [0.86; 1.04]	na	na	na
vs. supervised resistance						
unsupervised resistance	0	0	0.98 [0.86; 1.12]	na	na	na

NOTE: NA: not available; prop: direct evidence proportion; NMA: network meta-analysis

Appendix 5-10 Subgroup analysis of age in weight

Comparisons	Age＜60 (years)			Age≥60 (years)		
	No. of direct comparisons	prop	NMA 95%-CI	No. of direct comparisons	prop	NMA 95%-CI
vs. Combined						
no exercise	2	0.56	1.07 [1.01; 1.13]	na	na	na
supervised aerobic	1	0.26	1.05 [0.99; 1.11]	na	na	na
supervised resistance	1	0.31	1.05 [0.99; 1.11]	na	na	na
unsupervised aerobic	0	0	1.11 [1.04; 1.19]	na	na	na
unsupervised resistance	0	0	1.03 [0.96; 1.12]	na	na	na
vs. no exercise						
supervised aerobic	3	0.56	0.98 [0.95; 1.01]	3	1	1.01 [0.93; 1.08]
supervised resistance	3	0.53	0.98 [0.94; 1.02]	na	na	na
unsupervised aerobic	1	0.34	1.04 [1.00; 1.09]	na	na	na
unsupervised resistance	1	0.76	0.97 [0.91; 1.03]	na	na	na
vs. supervised aerobic						
supervised resistance	4	0.63	1.00 [0.97; 1.04]	na	na	na
unsupervised aerobic	1	0.7	1.06 [1.02; 1.11]	na	na	na
unsupervised resistance	0	0	0.99 [0.93; 1.06]	na	na	na
vs. supervised resistance						
unsupervised aerobic	0	0	1.06 [1.01; 1.12]	na	na	na
unsupervised resistance	0	0	0.99 [0.92; 1.06]	na	na	na
vs. unsupervised aerobic						
unsupervised resistance	1	0.36	0.93 [0.87; 0.99]	na	na	na
vs. anaerobic						
no exercise	na	na	na	0	0	1.04 [0.93; 1.17]
supervised aerobic	na	na	na	3	1	1.05 [0.97; 1.14]

NOTE: NA: not available; prop: direct evidence proportion; NMA: network meta-analysis

Appendix 5-11 Subgroup analysis of study duration in weight

Comparisons	Study duration ≤6 (month)			diabetes duration ＞6 (month)		
	No. of direct comparisons	prop	NMA 95%-CI	No. of direct comparisons	prop	NMA 95%-CI
vs. Combined						
flexibility training	na	na	na	na	na	na
no exercise	1	1	1.09 [0.98; 1.22]	1	0.49	1.04 [0.97; 1.12]
supervised aerobic	0	0	1.08 [0.96; 1.20]	1	0.42	1.06 [0.98; 1.14]
supervised resistance	0	0	1.07 [0.95; 1.20]	1	0.5	1.05 [0.97; 1.13]
unsupervised aerobic	0	0	1.09 [0.96; 1.24]	0	0	**1.15 [1.05; 1.26]**
unsupervised resistance	0	0	1.05 [0.92; 1.18]	na	na	na
vs. no exercise						
supervised aerobic	4	0.76	0.98 [0.95; 1.02]	2	0.67	1.02 [0.96; 1.08]
supervised resistance	2	0.55	0.98 [0.94; 1.02]	1	0.5	1.01 [0.94; 1.08]
unsupervised aerobic	1	0.74	1.00 [0.94; 1.07]	0	0	**1.11 [1.02; 1.20]**
unsupervised resistance	1	0.8	0.96 [0.90; 1.02]	na	na	na
vs. supervised aerobic						
supervised resistance	3	0.69	1.00 [0.96; 1.04]	1	0.43	0.99 [0.92; 1.07]
unsupervised aerobic	0	0	1.02 [0.94; 1.10]	1	1	**1.09 [1.04; 1.14]**
unsupervised resistance	0	0	0.97 [0.91; 1.04]	na	na	na
vs. supervised resistance						
unsupervised aerobic	0	0	1.02 [0.94; 1.10]	0	0	**1.10 [1.00; 1.20]**
unsupervised resistance	0	0	0.98 [0.91; 1.05]	na	na	na
vs. unsupervised aerobic						
unsupervised resistance	1	0.46	0.96 [0.89; 1.03]	na	na	na
vs. anaerobic						
combined	0	0	0.98 [0.85; 1.12]	na	na	na
no exercise	0	0	1.07 [0.98; 1.17]	na	na	na
supervised aerobic	3	1	1.05 [0.97; 1.14]	na	na	na
supervised resistance	0	0	1.05 [0.96; 1.15]	na	na	na
unsupervised aerobic	0	0	1.07 [0.95; 1.19]	na	na	na
unsupervised resistance	0	0	1.02 [0.92; 1.14]	na	na	na

NOTE: NA: not available; prop: direct evidence proportion; NMA: network meta-analysis

Appendix 5-12 Subgroup analysis of type of patients in weight

Comparisons	Sedentary			Non-sedentary		
	No. of direct comparisons	prop	NMA 95%-CI	No. of direct comparisons	prop	NMA 95%-CI
vs. Combined						
flexibility training	na	na	na	na	na	na
no exercise	1	0.44	1.04 [0.98; 1.11]	1	1	1.09 [0.98; 1.22]
supervised aerobic	1	0.38	1.06 [0.99; 1.13]	0	0	1.06 [0.94; 1.19]
supervised resistance	1	0.46	1.05 [0.97; 1.12]	0	0	1.06 [0.94; 1.19]
unsupervised aerobic	0	0	1.03 [0.93; 1.14]	0	0	1.12 [0.95; 1.32]
unsupervised resistance	na	na	na	0	0	1.05 [0.93; 1.19]
vs. no exercise						
supervised aerobic	3	0.81	1.01 [0.97; 1.06]	3	0.67	0.97 [0.92; 1.01]
supervised resistance	2	0.58	1.00 [0.95; 1.06]	1	0.56	0.97 [0.92; 1.02]
unsupervised aerobic	1	1	0.99 [0.92; 1.07]	0	0	1.02 [0.90; 1.16]
unsupervised resistance	na	na	na	1	1	0.96 [0.90; 1.03]
vs. supervised aerobic						
supervised resistance	1	0.33	0.99 [0.93; 1.05]	3	0.76	1.00 [0.96; 1.05]
unsupervised aerobic	0	0	0.98 [0.89; 1.07]	0	0	1.06 [0.92; 1.21]
unsupervised resistance	na	na	na	0	0	1.00 [0.92; 1.08]
vs. supervised resistance						
unsupervised aerobic	0	0	0.99 [0.90; 1.09]	0	0	1.05 [0.92; 1.21]
unsupervised resistance	na	na	na	0	0	0.99 [0.91; 1.08]
vs. unsupervised aerobic						
unsupervised resistance	na	na	na	1	1	0.94 [0.84; 1.05]
vs. flexibility training						
no exercise	na	na	na	na	na	na
supervised aerobic	na	na	na	na	na	na
supervised resistance	na	na	na	na	na	na
vs. anaerobic						
combined	0	0	1.00 [0.89; 1.13]	0	0	0.98 [0.82; 1.17]
no exercise	0	0	1.05 [0.94; 1.17]	0	0	1.07 [0.92; 1.23]
supervised aerobic	2	1	1.06 [0.96; 1.17]	1	1	1.03 [0.90; 1.18]
supervised resistance	0	0	1.05 [0.93; 1.18]	0	0	1.03 [0.90; 1.19]
unsupervised aerobic	0	0	1.04 [0.90; 1.19]	0	0	1.09 [0.90; 1.33]
unsupervised resistance	na	na	na	0	0	1.03 [0.88; 1.21]

NOTE: NA: not available; prop: direct evidence proportion; NMA: network meta-analysis

Appendix 5-13 Subgroup analysis of diabetes duration in SBP

Comparisons	diabetes duration ≤6 (years)			diabetes duration ＞6 (years)		
	No. of direct comparisons	prop	NMA 95%-CI	No. of direct comparisons	prop	NMA 95%-CI
vs. Combined						
no exercise	1	0.4	1.03 [0.98; 1.08]	1	1	1.00 [0.89; 1.12]
supervised aerobic	1	0.41	1.01 [0.96; 1.06]	0	0	0.95 [0.83; 1.09]
supervised resistance	1	0.41	0.99 [0.94; 1.04]	0	0	0.93 [0.80; 1.09]
unsupervised aerobic	0	0	1.02 [0.94; 1.09]	na	na	na
vs. no exercise						
supervised aerobic	4	0.75	0.98 [0.95; 1.01]	2	1	0.95 [0.88; 1.02]
supervised resistance	3	0.63	0.97 [0.93; 1.00]	0	0	0.93 [0.84; 1.04]
unsupervised aerobic	1	0.54	0.99 [0.93; 1.05]	na	na	na
vs. supervised aerobic						
supervised resistance	2	0.32	0.98 [0.94; 1.02]	2	1	0.98 [0.91; 1.06]
unsupervised aerobic	0	0	1.01 [0.94; 1.07]	na	na	na
vs. supervised resistance						
unsupervised aerobic	1	0.55	1.02 [0.96; 1.08]	na	na	na

NOTE: NA: not available; prop: direct evidence proportion; NMA: network meta-analysis; SBP: systolic blood pressure

Appendix 5-14 Subgroup analysis of age in SBP

Comparisons	Age＜60 (years)			Age≥60 (years)		
	No. of direct comparisons	prop	NMA 95%-CI	No. of direct comparisons	prop	NMA 95%-CI
vs. Combined						
no exercise	1	0.39	1.03 [0.98; 1.08]	1	1	1.00 [0.92; 1.09]
supervised aerobic	1	0.38	1.01 [0.96; 1.06]	0	0	0.89 [0.79; 1.01]
supervised resistance	1	0.38	0.99 [0.95; 1.04]	1	1	1.08 [0.96; 1.22]
unsupervised aerobic	0	0	0.99 [0.93; 1.05]	na	na	na
unsupervised resistance	0	0	0.97 [0.88; 1.07]	na	na	na
vs. no exercise						
supervised aerobic	5	0.72	0.98 [0.95; 1.01]	1	1	**0.90 [0.82; 0.98]**
supervised resistance	3	0.53	0.96 [0.93; 0.99]	na	na	na
unsupervised aerobic	2	0.73	0.96 [0.92; 1.00]	0	0	1.08 [0.94; 1.25]
unsupervised resistance	0	0	0.94 [0.86; 1.03]	na	na	na
vs. supervised aerobic						
supervised resistance	4	0.5	0.98 [0.95; 1.01]	na	na	na
unsupervised aerobic	0	0	0.98 [0.93; 1.03]	0	0	**1.21 [1.02; 1.43]**
unsupervised resistance	0	0	0.96 [0.87; 1.06]	na	na	na
vs. supervised resistance						
unsupervised aerobic	1	0.37	1.00 [0.95; 1.04]	na	na	na
unsupervised resistance	0	0	0.98 [0.89; 1.08]	na	na	na
vs. unsupervised aerobic						
unsupervised resistance	1	1	0.98 [0.91; 1.07]	na	na	na
vs. anaerobic						
no exercise	na	na	na	0	0	1.10 [0.95; 1.27]
supervised aerobic	na	na	na	0	0	1.10 [0.98; 1.24]
supervised resistance	na	na	na	2	1	0.99 [0.91; 1.07]
unsupervised aerobic	na	na	na	0	0	1.19 [0.99; 1.44]

NOTE: NA: not available; prop: direct evidence proportion; NMA: network meta-analysis; SBP: systolic blood pressure

Appendix 5-14 Subgroup analysis of study duration in SBP

Comparisons	Study duration ≤6 (month)			Study duration ＞6 (month)		
	No. of direct comparisons	prop	NMA 95%-CI	No. of direct comparisons	prop	NMA 95%-CI
vs. Combined						
flexibility training	na	na	na	na	na	na
no exercise	1	0.67	1.04 [0.97; 1.12]	1	0.48	1.01 [0.96; 1.07]
supervised aerobic	0	0	1.01 [0.93; 1.09]	1	0.47	1.00 [0.95; 1.06]
supervised resistance	0	0	0.98 [0.90; 1.06]	1	0.49	1.02 [0.96; 1.07]
unsupervised aerobic	1	0.39	1.00 [0.92; 1.08]	na	na	na
unsupervised resistance	0	0	0.98 [0.88; 1.09]	na	na	na
vs. no exercise						
supervised aerobic	4	0.82	0.97 [0.94; 1.00]	2	0.62	0.99 [0.94; 1.04]
supervised resistance	2	0.55	0.94 [0.91; 0.97]	1	0.48	1.00 [0.95; 1.06]
unsupervised aerobic	2	0.7	0.96 [0.92; 1.00]	na	na	na
unsupervised resistance	0	0	0.94 [0.86; 1.03]	na	na	na
vs. supervised aerobic						
supervised resistance	3	0.48	0.97 [0.94; 1.00]	1	0.46	1.02 [0.96; 1.07]
unsupervised aerobic	0	0	0.99 [0.94; 1.04]	na	na	na
unsupervised resistance	0	0	0.97 [0.89; 1.06]	na	na	na
vs. supervised resistance						
unsupervised aerobic	1	0.38	1.02 [0.98; 1.07]	na	na	na
unsupervised resistance	0	0	1.00 [0.92; 1.09]	na	na	na
vs. unsupervised aerobic						
unsupervised resistance	1	1	0.98 [0.91; 1.06]	na	na	na
vs. anaerobic						
combined	0	0	0.98 [0.87; 1.09]	na	na	na
no exercise	0	0	1.02 [0.93; 1.11]	na	na	na
supervised aerobic	2	1	0.99 [0.91; 1.07]	na	na	na
supervised resistance	0	0	0.96 [0.87; 1.05]	na	na	na
unsupervised aerobic	0	0	0.97 [0.89; 1.07]	na	na	na
unsupervised resistance	0	0	0.96 [0.85; 1.08]	na	na	na

NOTE: NA: not available; prop: direct evidence proportion; NMA: network meta-analysis; SBP: systolic blood pressure

Appendix 5-15 Subgroup analysis of type of patients in SBP

Comparisons	Sedentary			Non-sedentary		
	No. of direct comparisons	prop	NMA 95%-CI	No. of direct comparisons	prop	NMA 95%-CI
vs. Combined						
flexibility training	na	na	na	na	na	na
no exercise	na	na	na	2	0.47	1.03 [0.99; 1.07]
supervised aerobic	na	na	na	1	0.33	1.01 [0.97; 1.05]
supervised resistance	na	na	na	1	0.34	0.99 [0.95; 1.03]
unsupervised aerobic	na	na	na	1	0.21	1.02 [0.97; 1.08]
unsupervised resistance	na	na	na	0	0	1.01 [0.91; 1.11]
vs. no exercise						
supervised aerobic	2	0.65	0.93 [0.86; 1.01]	4	0.78	0.98 [0.96; 1.01]
supervised resistance	1	0.61	0.96 [0.88; 1.04]	2	0.55	**0.96 [0.93; 0.99]**
unsupervised aerobic	1	1	0.92 [0.84; 1.00]	1	0.47	0.99 [0.95; 1.05]
unsupervised resistance	na	na	na	0	0	0.98 [0.89; 1.07]
vs. supervised aerobic						
supervised resistance	1	0.74	1.03 [0.96; 1.11]	3	0.36	0.98 [0.94; 1.01]
unsupervised aerobic	0	0	0.98 [0.87; 1.11]	0	0	1.01 [0.96; 1.07]
unsupervised resistance	na	na	na	0	0	1.00 [0.90; 1.09]
vs. supervised resistance						
unsupervised aerobic	0	0	0.96 [0.85; 1.08]	1	0.48	1.04 [0.98; 1.09]
unsupervised resistance	na	na	na	0	0	1.02 [0.93; 1.12]
vs. unsupervised aerobic						
unsupervised resistance	na	na	na	0	0	0.98 [0.91; 1.06]
vs. flexibility training						
no exercise	na	na	na	na	na	na
supervised aerobic	na	na	na	na	na	na
supervised resistance	na	na	na	na	na	na
vs. anaerobic						
combined				0	0	0.97 [0.85; 1.10]
no exercise	0	0	1.07 [0.91; 1.25]	0	0	1.00 [0.88; 1.12]
supervised aerobic	1	1	0.99 [0.87; 1.14]	1	1	0.98 [0.87; 1.10]
supervised resistance	0	0	1.02 [0.88; 1.19]	0	0	0.96 [0.84; 1.08]
unsupervised aerobic	0	0	0.98 [0.82; 1.17]	0	0	0.99 [0.87; 1.13]
unsupervised resistance	na	na	na	0	0	0.97 [0.84; 1.13]

NOTE: NA: not available; prop: direct evidence proportion; NMA: network meta-analysis; SBP: systolic blood pressure

Appendix 5-16 Subgroup analysis of diabetes duration in DBP

Comparisons	diabetes duration ≤6 (years)			diabetes duration ＞6 (years)		
	No. of direct comparisons	prop	NMA 95%-CI	No. of direct comparisons	prop	NMA 95%-CI
vs. Combined						
flexibility training	na	na	na	na	na	na
no exercise	1	0.42	1.01 [0.96; 1.07]	1	1	0.97 [0.88; 1.08]
supervised aerobic	1	0.41	0.98 [0.93; 1.03]	0	0	0.96 [0.86; 1.07]
supervised resistance	1	0.44	1.00 [0.94; 1.06]	0	0	0.97 [0.86; 1.10]
vs. no exercise						
supervised aerobic	4	0.76	0.97 [0.93; 1.01]	2	1	0.99 [0.95; 1.03]
supervised resistance	2	0.54	0.99 [0.95; 1.04]	0	0	1.00 [0.93; 1.07]
vs. supervised aerobic						
supervised resistance	2	0.43	1.02 [0.97; 1.07]	2	1	1.01 [0.95; 1.07]

NOTE: NA: not available; prop: direct evidence proportion; NMA: network meta-analysis; DBP: diastolic blood pressure

Appendix 6-14 subgroup analysis of age in DBP

Comparisons	Age＜60 (years)			Age≥60 (years)		
	No. of direct comparisons	prop	NMA 95%-CI	No. of direct comparisons	prop	NMA 95%-CI
vs. Combined						
no exercise	1	0.39	1.02 [0.96; 1.08]	1	1	0.97 [0.88; 1.08]
supervised aerobic	1	0.38	0.98 [0.93; 1.05]	0	0	0.97 [0.85; 1.10]
supervised resistance	1	0.39	0.98 [0.92; 1.05]	na	na	na
unsupervised aerobic	0	0	0.96 [0.89; 1.04]	na	na	na
unsupervised resistance	0	0	0.99 [0.87; 1.13]	na	na	na
vs. no exercise						
supervised aerobic	5	0.7	0.96 [0.93; 1.00]	1	1	1.00 [0.92; 1.07]
supervised resistance	3	0.51	0.96 [0.93; 1.00]	na	na	na
unsupervised aerobic	2	0.74	0.94 [0.89; 1.00]	na	na	na
unsupervised resistance	0	0	0.97 [0.86; 1.09]	na	na	na
vs. supervised aerobic						
supervised resistance	4	0.52	1.00 [0.96; 1.04]	na	na	na
unsupervised aerobic	0	0	0.98 [0.92; 1.05]	na	na	na
unsupervised resistance	0	0	1.01 [0.89; 1.14]	na	na	na
vs. supervised resistance						
unsupervised aerobic	1	0.36	0.98 [0.92; 1.04]	na	na	na
unsupervised resistance	0	0	1.01 [0.89; 1.14]	na	na	na
vs. unsupervised aerobic						
unsupervised resistance	1	1	1.03 [0.92; 1.14]	na	na	na
vs. anaerobic						
combined	na	na	na	0	0	0.96 [0.83; 1.10]
no exercise	na	na	na	0	0	0.93 [0.85; 1.02]
supervised aerobic	na	na	na	2	1	0.92 [0.88; 0.97]

NOTE: NA: not available; prop: direct evidence proportion; NMA: network meta-analysis; DBP: diastolic blood pressure

Appendix 6-15 subgroup analysis of study duration in DBP

Comparisons	Study duration ≤6 (month)			Study duration ＞6 (month)		
	No. of direct comparisons	prop	NMA 95%-CI	No. of direct comparisons	prop	NMA 95%-CI
vs. Combined						
no exercise	1	1	0.97 [0.85; 1.12]	1	0.48	1.00 [0.96; 1.04]
supervised aerobic	0	0	0.93 [0.80; 1.08]	1	0.46	0.99 [0.95; 1.03]
supervised resistance	0	0	0.92 [0.79; 1.07]	1	0.49	1.00 [0.96; 1.04]
unsupervised aerobic	0	0	0.91 [0.79; 1.07]	na	na	na
unsupervised resistance	0	0	0.94 [0.78; 1.14]	na	na	na
vs. no exercise						
supervised aerobic	4	0.77	0.96 [0.91; 1.01]	2	0.63	0.99 [0.95; 1.03]
supervised resistance	2	0.51	0.95 [0.90; 1.00]	1	0.48	1.00 [0.96; 1.04]
unsupervised aerobic	2	0.75	0.94 [0.88; 1.00]	na	na	na
unsupervised resistance	0	0	0.97 [0.85; 1.10]	na	na	na
vs. supervised aerobic						
supervised resistance	3	0.56	0.99 [0.93; 1.05]	1	0.46	1.01 [0.97; 1.06]
unsupervised aerobic	0	0	0.98 [0.91; 1.06]	na	na	na
unsupervised resistance	0	0	1.01 [0.88; 1.16]	na	na	na
vs. supervised resistance						
unsupervised aerobic	1	0.41	0.99 [0.92; 1.07]	na	na	na
unsupervised resistance	0	0	1.02 [0.89; 1.17]	na	na	na
vs. unsupervised aerobic						
unsupervised resistance	1	1	1.03 [0.91; 1.15]	na	na	na
vs. anaerobic						
combined	0	0	1.00 [0.84; 1.19]	na	na	na
no exercise	0	0	0.97 [0.88; 1.08]	na	na	na
supervised aerobic	2	1	0.93 [0.85; 1.02]	na	na	na
supervised resistance	0	0	0.92 [0.83; 1.02]	na	na	na
unsupervised aerobic	0	0	0.92 [0.81; 1.03]	na	na	na
unsupervised resistance	0	0	0.94 [0.80; 1.11]	na	na	na

NOTE: NA: not available; prop: direct evidence proportion; NMA: network meta-analysis; DBP: diastolic blood pressure

Appendix 5- Subgroup analysis of type of patients in DBP

Comparisons	Sedentary			Non-sedentary		
	No. of direct comparisons	prop	NMA 95%-CI	No. of direct comparisons	prop	NMA 95%-CI
vs. Combined						
flexibility training	na	na	na	na	na	na
no exercise	na	na	na	2	0.49	1.01 [0.97; 1.06]
supervised aerobic	na	na	na	1	0.35	0.99 [0.94; 1.04]
supervised resistance	na	na	na	1	0.38	1.02 [0.97; 1.07]
unsupervised aerobic	na	na	na	0	0	0.99 [0.92; 1.07]
unsupervised resistance	na	na	na	0	0	1.03 [0.92; 1.16]
vs. no exercise						
supervised aerobic	1	0.32	0.98 [0.89; 1.06]	5	0.77	0.97 [0.94; 1.00]
supervised resistance	1	0.83	1.01 [0.94; 1.08]	2	0.50	0.96 [0.93; 1.00]
unsupervised aerobic	0	0	**0.88 [0.84; 0.92]**	1	0.56	0.99 [0.93; 1.06]
unsupervised resistance	na	na	na	0	0	1.02 [0.91; 1.14]
vs. supervised aerobic						
supervised resistance	1	0.85	1.03 [0.97; 1.10]	3	0.43	1.01 [0.97; 1.05]
unsupervised aerobic	0	0	**1.11 [1.01; 1.23]**	0	0	0.98 [0.91; 1.05]
unsupervised resistance	na	na	na	0	0	0.95 [085; 1.07]
vs. supervised resistance						
unsupervised aerobic	0	0	**1.15 [1.06; 1.25]**	1	0.53	0.97 [0.91; 1.03]
unsupervised resistance	na	na	na	0	0	0.94 [0.84; 1.06]
vs. unsupervised aerobic						
unsupervised resistance	na	na	na	1	1	0.97 [0.89; 1.07]
vs. anaerobic						
combined	na	na	na	0	0	1.07 [0.98; 1.17]
no exercise	0	0	1.01 [0.87; 1.18]	0	0	1.06 [0.97; 1.15]
supervised aerobic	1	1	1.04 [0.91; 1.18]	1	1	1.09 [1.01; 1.18]
supervised resistance	0	0	1.01 [0.87; 1.16]	0	0	1.10 [1.01; 1.20]
unsupervised aerobic	0	0	1.16 [0.98; 1.36]	0	0	1.06 [0.96; 1.18]
unsupervised resistance	na	na	na	0	0	1.04 [0.90; 1.19]

NOTE: NA: not available; prop: direct evidence proportion; NMA: network meta-analysis; DBP: diastolic blood pressure

Appendix 6-16 subgroup analysis of diabetes duration in TC

Comparisons	diabetes duration ≤6 (years)			diabetes duration ＞6 (years)		
	No. of direct comparisons	prop	NMA 95%-CI	No. of direct comparisons	prop	NMA 95%-CI
vs. Combined						
flexibility training	na	na	na	1	0.44	0.93 [0.83; 1.05]
no exercise	na	na	na	2	0.75	0.99 [0.92; 1.07]
supervised aerobic	na	na	na	1	0.25	0.88 [0.80; 0.96]
supervised resistance	na	na	na	1	0.38	0.87 [0.78; 0.97]
vs. no exercise						
supervised aerobic	1	0.52	0.91 [0.80; 1.04]	2	0.69	**0.88 [0.81; 0.96]**
supervised resistance	1	0.61	0.86 [0.79; 0.93]	0	0	0.88 [0.78; 0.98]
unsupervised aerobic	1	0.62	0.91 [0.84; 0.98]	na	na	na
vs. supervised aerobic						
supervised resistance	1	0.57	0.94 [0.82; 1.07]	2	0.64	
unsupervised aerobic	0	0	0.99 [0.86; 1.14]	na	na	na
vs. supervised resistance						
unsupervised aerobic	1	0.68	1.06 [0.98; 1.14]	na	na	na
vs. flexibility training						
no exercise	na	na	na	0	0	1.07 [0.94; 1.21]
supervised aerobic	na	na	na	1	0.4	0.94 [0.84; 1.06]
supervised resistance	na	na	na	1	0.45	0.94 [0.83; 1.05]

NOTE: NA: not available; prop: direct evidence proportion; NMA: network meta-analysis; TC: total cholesterol

Appendix 6-17 subgroup analysis of age in TC

Comparisons	Age＜60 (years)			Age≥60 (years)		
	No. of direct comparisons	prop	NMA 95%-CI	No. of direct comparisons	prop	NMA 95%-CI
vs. Combined						
flexibility training	1	0.45	0.96 [0.86; 1.06]			
no exercise	1	0.42	1.05 [0.96; 1.14]	1	1	0.92 [0.85; 1.00]
supervised aerobic	1	0.3	0.92 [0.85; 1.00]	0	0	**0.81 [0.72; 0.92]**
supervised resistance	1	0.32	0.91 [0.84; 0.99]	na	na	na
unsupervised aerobic	0	0	1.00 [0.91; 1.10]	na	na	na
vs. no exercise						
supervised aerobic	2	0.48	0.88 [0.83; 0.94]	2	1	0.88 [0.81; 0.96]
supervised resistance	1	0.39	0.87 [0.82; 0.92]	na	na	na
unsupervised aerobic	2	0.7	0.95 [0.90; 1.01]	na	na	na
vs. supervised aerobic						
supervised resistance	3	0.53	0.98 [0.92; 1.05]	na	na	na
unsupervised aerobic	2	0.1	1.08 [1.00; 1.17]	na	na	na
vs. supervised resistance						
unsupervised aerobic	1	0.52	1.10 [1.02; 1.17]	na	na	na
vs. flexibility training						
no exercise	0	0	1.09 [0.98; 1.21]	na	na	na
supervised aerobic	1	0.39	0.96 [0.87; 1.06]	na	na	na
supervised resistance	1	0.41	0.95 [0.86; 1.05]	na	na	na
unsupervised aerobic	0	0	1.04 [0.93; 1.16]	na	na	na

NOTE: NA: not available; prop: direct evidence proportion; NMA: network meta-analysis; TC: total cholesterol

Appendix 6-18 subgroup analysis of study duration in TC

Comparisons	Study duration ≤6 (month)			diabetes duration ＞6 (month)		
	No. of direct comparisons	prop	NMA 95%-CI	No. of direct comparisons	prop	NMA 95%-CI
vs. Combined						
flexibility training	1	0.41	0.93 [0.84; 1.03]	na	na	na
no exercise	2	0.73	0.97 [0.92; 1.03]	na	na	na
supervised aerobic	1	0.21	0.89 [0.83; 0.95]	na	na	na
supervised resistance	1	0.23	0.86 [0.80; 0.93]	na	na	na
unsupervised aerobic	0	0	0.93 [0.85; 1.01]	na	na	na
vs. no exercise						
supervised aerobic	3	0.59	0.91 [0.86; 0.97]	1	1	0.85 [0.76; 0.94]
supervised resistance	1	0.33	0.89 [0.84; 0.94]	na	na	na
unsupervised aerobic	2	0.71	0.95 [0.89; 1.02]	0	0	1.02 [0.77; 1.34]
vs. supervised aerobic						
supervised resistance	3	0.52	0.97 [0.91; 1.04]	na	na	na
unsupervised aerobic	0	0	1.05 [0.96; 1.14]	2	1	1.20 [0.93; 1.55]
vs. supervised resistance						
unsupervised aerobic	1	0.51	1.07 [1.00; 1.16]	na	na	na
vs. flexibility training						
no exercise	0	0	1.05 [0.94; 1.16]	na	na	na
supervised aerobic	1	0.38	0.95 [0.86; 1.06]	na	na	na
supervised resistance	1	0.40	0.93 [0.84; 1.03]	na	na	na

NOTE: NA: not available; prop: direct evidence proportion; NMA: network meta-analysis; TC: total cholesterol

Appendix 5- Subgroup analysis of type of patients in TC

Comparisons	Sedentary			Non-sedentary		
	No. of direct comparisons	prop	NMA 95%-CI	No. of direct comparisons	prop	NMA 95%-CI
vs. Combined						
flexibility training	na	na	na	1	0.41	0.92 [0.83; 1.02]
no exercise	na	na	na	2	0.79	0.97 [0.92; 1.02]
supervised aerobic	na	na	na	1	0.25	0.88 [0.82; 0.95]
supervised resistance	na	na	na	1	0.23	0.84 [0.78; 0.90]
unsupervised aerobic	na	na	na	0	0	0.89 [0.82; 0.97]
unsupervised resistance	na	na	na	na	na	na
vs. no exercise						
supervised aerobic	3	1	0.89 [0.83; 0.95]	1	0.41	0.91 [0.85; 0.98]
supervised resistance	0	0	0.96 [0.83; 1.11]	1	0.42	**0.86 [0.81; 0.92]**
unsupervised aerobic	1	1	1.03 [0.93; 1.14]	1	0.53	**0.92 [0.85; 0.99]**
unsupervised resistance	na	na	na	na	na	na
vs. supervised aerobic						
supervised resistance	1	1	1.08 [0.95; 1.23]	2	0.42	0.95 [0.88; 1.02]
unsupervised aerobic	0	0	1.16 [1.02; 1.31]	2	0.13	1.01 [0.92; 1.11]
unsupervised resistance	na	na	na	na	na	na
vs. supervised resistance						
unsupervised aerobic	0	0	1.08 [0.90; 1.29]	1	0.61	1.07 [0.99; 1.15]
unsupervised resistance	na	na	na	na	na	na
vs. unsupervised aerobic						
unsupervised resistance	na	na	na	na	na	na
vs. flexibility training						
no exercise	na	na	na	0	0	1.05 [0.95; 1.17]
supervised aerobic	na	na	na	1	0.4	0.96 [0.87; 1.06]
supervised resistance	na	na	na	1	0.41	0.91 [0.83; 1.01]

NOTE: NA: not available; prop: direct evidence proportion; NMA: network meta-analysis; TC: total cholesterol

Appendix 6-19 subgroup analysis of diabetes duration in TG

Comparisons	diabetes duration ≤6 (years)			diabetes duration ＞6 (years)		
	No. of direct comparisons	prop	NMA 95%-CI	No. of direct comparisons	prop	NMA 95%-CI
vs. Combined						
no exercise	2	0.43	**1.40 [1.22; 1.61]**	2	1	1.17 [0.94; 1.46]
supervised aerobic	2	0.47	**1.24 [1.07; 1.44]**	0	0	1.00 [0.77; 1.30]
supervised resistance	2	0.41	**1.21 [1.05; 1.39]**	0	0	1.94 [0.93; 4.03]
unsupervised aerobic	0	0	1.28 [1.09; 1.52]	na	na	na
vs. no exercise						
supervised aerobic	3	0.5	0.88 [0.78; 1.01]	2	1	0.85 [0.74; 0.98]
supervised resistance	3	0.56	**0.86 [0.78; 0.95]**	0	0	1.66 [0.83; 3.33]
unsupervised aerobic	1	0.51	0.92 [0.81; 1.03]	na	na	na
vs. supervised aerobic						
supervised resistance	3	0.41	0.97 [0.85; 1.12]	1	1	1.94 [0.98; 3.85]
unsupervised aerobic	0	0	1.04 [0.88; 1.22]	na	na	na
vs. supervised resistance						
unsupervised aerobic	1	0.7	1.06 [0.95; 1.18]	na	na	na

NOTE: NA: not available; prop: direct evidence proportion; NMA: network meta-analysis; TG: triacylglycerol

Appendix 6-20 subgroup analysis of age in TG

Comparisons	Age＜60 (years)			Age≥60 (years)		
	No. of direct comparisons	prop	NMA 95%-CI	No. of direct comparisons	prop	NMA 95%-CI
vs. combined						
flexibility training						
no exercise	3	0.56	**1.35 [1.20; 1.52]**	1	1	0.98 [0.57; 1.69]
supervised aerobic	2	0.38	**1.19 [1.04; 1.37]**	0	0	0.82 [0.47; 1.46]
supervised resistance	2	0.34	1.20 [1.05; 1.36]	na	na	na
unsupervised aerobic	0	0	1.29 [1.11; 1.49]	na	na	na
vs. no exercise						
supervised aerobic	5	0.61	**0.88 [0.79; 0.98]**	1	1	0.84 [0.71; 1.00]
supervised resistance	3	0.5	0.89 [0.81; 0.97]	na	na	na
unsupervised aerobic	2	0.62	0.95 [0.86; 1.06]	na	na	na
vs. supervised aerobic						
supervised resistance	4	0.36	1.00 [0.89; 1.13]	na	na	na
unsupervised aerobic	1	0.01	1.08 [0.94; 1.24]	na	na	na
vs. supervised resistance						
unsupervised aerobic	1	0.63	1.08 [0.97; 1.19]	na	na	na

NOTE: NA: not available; prop: direct evidence proportion; NMA: network meta-analysis; TG: triacylglycerol

Appendix 6-21 subgroup analysis of study duration in TG

Comparisons	Study duration ≤6 (month)			Study duration ＞6 (month)		
	No. of direct comparisons	prop	NMA 95%-CI	No. of direct comparisons	prop	NMA 95%-CI
vs. Combined						
no exercise	2	1.00	1.17 [0.94; 1.46]			
supervised aerobic	0	0	1.02 [0.77; 1.35]	2	0.43	**1.21 [1.05; 1.41]**
supervised resistance	0	0	1.03 [0.80; 1.33]	2	0.49	**1.23 [1.05; 1.43]**
unsupervised aerobic	0	0	1.11 [0.87;1.42]	0	0	2.31 [0.42; 12.81]
vs. no exercise						
supervised aerobic	3	0.86	0.87 [0.74; 1.04]	3	0.73	**0.86 [0.77; 0.96]**
supervised resistance	1	0.52	**0.88 [0.78; 0.99]**	2	0.45	0.87 [0.75; 1.00]
unsupervised aerobic	2	0.70	0.95 [0.85;1.06]	0	0	1.63 [0.30; 9.04]
vs. supervised aerobic						
supervised resistance	2	0.20	1.01 [0.83; 1.23]	2	0.43	1.01 [0.87; 1.17]
unsupervised aerobic	0	0	1.08 [0.89; 1.32]	1	1	1.90 [0.34; 10.48]
vs. supervised resistance						
unsupervised aerobic	1	0.71	1.08 [0.96;1.20]	0	0	1.88 [0.34; 10.45]

NOTE: NA: not available; prop: direct evidence proportion; NMA: network meta-analysis; TG: triacylglycerol

Appendix 5- Subgroup analysis of type of patients in TG

Comparisons	Sedentary				Non-sedentary			
	No. of direct comparisons	prop	NMA	95%-CI	No. of direct comparisons	prop	NMA	95%-CI
vs. Combined								
no exercise	1	0.43	1.40	[1.14; 1.70]	3	0.66	1.31	[1.13; 1.51]
supervised aerobic	1	0.39	1.22	[1.00; 1.49]	1	0.34	1.13	[0.95; 1.36]
supervised resistance	1	0.49	1.24	[1.00; 1.54]	1	0.26	1.14	[0.97; 1.34]
unsupervised aerobic	0	0	1.45	[1.08; 1.93]	0	0	1.21	[1.01; 1.44]
vs. no exercise								
supervised aerobic	4	0.84	0.87	[0.77; 0.98]	2	0.60	0.87	[0.75; 1.00]
supervised resistance	1	0.41	0.89	[0.73; 1.08]	2	0.55	**0.87**	**[0.78; 0.97]**
unsupervised aerobic	1	1	1.04	[0.84; 1.28]	1	0.54	0.92	[0.82; 1.04]
unsupervised resistance								
vs. supervised aerobic								
supervised resistance	2	0.44	1.02	[0.84; 1.24]	2	0.33	1.00	[0.85; 1.18]
unsupervised aerobic	0	0	1.19	[0.93; 1.52]	1	0.01	1.07	[0.89; 1.27]
vs. supervised resistance								
unsupervised aerobic	0	0	1.16	[0.87; 1.55]	1	0.72	1.06	[0.95; 1.18]

NOTE: NA: not available; prop: direct evidence proportion; NMA: network meta-analysis; TG: triacylglycerol

Appendix 6-22 subgroup analysis of diabetes duration in LDL

Comparisons	diabetes duration ≤6 (years)			diabetes duration ＞6 (years)		
	No. of direct comparisons	prop	NMA 95%-CI	No. of direct comparisons	prop	NMA 95%-CI
vs. combined						
flexibility training	na	na	na	1	0.46	0.91 [0.68; 1.22]
no exercise	1	0.44	1.03 [0.92; 1.14]	2	0.67	1.14 [0.91; 1.44]
supervised aerobic	1	0.42	0.98 [0.88; 1.10]	1	0.31	0.92 [0.73; 1.16]
supervised resistance	1	0.48	1.03 [0.92; 1.16]	1	0.41	0.85 [0.65; 1.11]
vs. no exercise						
supervised aerobic	2	0.8	0.96 [0.89; 1.03]	2	0.67	0.81 [0.64; 1.02]
supervised resistance	1	0.42	1.00 [0.90; 1.12]	0	0	0.74 [0.55; 1.00]
vs. supervised aerobic						
supervised resistance	2	0.44	1.05 [0.94; 1.16]	2	0.62	0.92 [0.72; 1.17]
vs. flexibility training						
no exercise	na	na	na	0	0	1.25 [0.90; 1.74]
supervised aerobic	na	na	na	1	0.41	1.01 [0.76; 1.34]
supervised resistance	na	na	na	1	0.45	0.93 [0.70; 1.24]

NOTE: NA: not available; prop: direct evidence proportion; NMA: network meta-analysis; LDL: low-density lipoprotein cholesterol

Appendix 6-23 subgroup analysis of age in LDL

Comparisons	Age＜60 (years)			Age≥60 (years)		
	No. of direct comparisons	prop	NMA 95%-CI	No. of direct comparisons	prop	NMA 95%-CI
vs. Combined						
flexibility training	1	0.42	0.98 [0.80; 1.20]	na	na	na
no exercise	2	0.48	1.13 [0.98; 1.29]	1	1	0.87 [0.72; 1.04]
supervised aerobic	2	0.38	1.00 [0.88; 1.14]	0	0	**0.70 [0.55; 0.90]**
supervised resistance	2	0.45	0.97 [0.85; 1.12]	na	na	na
unsupervised aerobic	0	0	1.21 [0.97; 1.52]	na	na	na
unsupervised resistance	0	0	1.26 [0.91; 1.76]	na	na	na
vs. no exercise						
supervised aerobic	4	0.64	0.89 [0.79; 0.99]	1	1	0.81 [0.68; 0.96]
supervised resistance	1	0.25	0.86 [0.75; 1.00]	na	na	na
unsupervised aerobic	1	0.48	1.08 [0.88; 1.31]	na	na	na
unsupervised resistance	0	0	1.12 [0.82; 1.54]	na	na	na
vs. supervised aerobic						
supervised resistance	4	0.56	0.97 [0.86; 1.11]	na	na	na
unsupervised aerobic	2	0.6	1.22 [1.00; 1.48]	na	na	na
unsupervised resistance	0	0	1.26 [0.92; 1.73]	na	na	na
vs. supervised resistance						
unsupervised aerobic	0	0	1.25 [0.99; 1.56]	na	na	na
unsupervised resistance	0	0	1.30 [0.93; 1.81]	na	na	na
vs. unsupervised aerobic						
unsupervised resistance	1	1	1.04 [0.81; 1.33]	na	na	na
vs. flexibility training						
no exercise	0	0	1.15 [0.93; 1.43]	na	na	na
supervised aerobic	1	0.36	1.02 [0.83; 1.25]	na	na	na
supervised resistance	1	0.39	1.00 [0.81; 1.22]	na	na	na
unsupervised aerobic	0	0	1.24 [0.94; 1.64]	na	na	na
unsupervised resistance	0	0	1.29 [0.89; 1.87]	na	na	na

NOTE: NA: not available; prop: direct evidence proportion; NMA: network meta-analysis; LDL: low-density lipoprotein cholesterol

Appendix 6-24 subgroup analysis of study duration in LDL

Comparisons	Study duration ≤6 (month)			Study duration ＞6 (month)		
	No. of direct comparisons	prop	NMA 95%-CI	No. of direct comparisons	prop	NMA 95%-CI
vs. combined						
flexibility training	1	0.46	0.93 [0.72; 1.20]	na	na	na
no exercise	2	0.62	1.11 [0.92; 1.35]	1	0.47	1.03 [0.92; 1.15]
supervised aerobic	1	0.28	0.95 [0.78; 1.16]	1	0.46	0.97 [0.87; 1.09]
supervised resistance	1	0.38	0.86 [0.69; 1.09]	1	0.49	1.03 [0.92; 1.16]
unsupervised aerobic	0	0	1.09 [0.73; 1.64]	0	0	**1.29 [1.06; 1.55]**
unsupervised resistance	0	0	1.14 [0.68; 1.90]	na	na	na
vs. no exercise						
supervised aerobic	3	0.77	0.86 [0.72; 1.02]	2	0.66	0.95 [0.86; 1.04]
supervised resistance	0	0	**0.78 [0.61; 0.99]**	1	0.47	1.00 [0.90; 1.12]
unsupervised aerobic	1	1	0.98 [0.69; 1.40]	0	0	**1.25 [1.04; 1.49]**
unsupervised resistance	0	0	1.02 [0.63; 1.64]	na	na	na
vs. supervised aerobic						
supervised resistance	3	0.66	0.91 [0.74; 1.11]	1	0.45	1.06 [0.95; 1.19]
unsupervised aerobic	0	0	1.15 [0.77; 1.70]	2	1.00	**1.32 [1.13; 1.54]**
unsupervised resistance	0	0	1.19 [0.72; 1.98]	na	na	na
vs. supervised resistance						
unsupervised aerobic	0	0	1.27 [0.82; 1.94]	0	0	**1.24 [1.03; 1.50]**
unsupervised resistance	0	0	1.31 [0.77; 2.24]	na	na	na
vs. unsupervised aerobic						
unsupervised resistance	1	1	1.04 [0.76; 1.43]	na	na	na
vs. flexibility training						
no exercise	0	0	1.20 [0.91; 1.59]	na	na	na
supervised aerobic	1	0.4	1.03 [0.80; 1.32]	na	na	na
supervised resistance	1	0.44	0.93 [0.72; 1.21]	na	na	na

NOTE: NA: not available; prop: direct evidence proportion; NMA: network meta-analysis; LDL: low-density lipoprotein cholesterol

Appendix 5- Subgroup analysis of type of patients in LDL

Comparisons	Sedentary			Non-sedentary		
	No. of direct comparisons	prop	NMA 95%-CI	No. of direct comparisons	prop	NMA 95%-CI
vs. Combined						
flexibility training	na	na	na	1	0.41	0.96 [0.75; 1.24]
no exercise	na	na	na	3	0.63	1.06 [0.90; 1.25]
supervised aerobic	na	na	na	2	0.41	0.99 [0.83; 1.17]
supervised resistance	na	na	na	2	0.46	0.94 [0.79; 1.12]
unsupervised aerobic	na	na	na	0	0	1.28 [0.91; 1.81]
unsupervised resistance	na	na	na	0	0	1.33 [0.83; 2.15]
vs. no exercise						
supervised aerobic	3	1	**0.89 [0.83; 0.96]**	2	0.50	0.93 [0.78; 1.11]
supervised resistance	0	0	0.96 [0.77; 1.20]	1	0.29	0.89 [0.73; 1.08]
unsupervised aerobic	1	1	0.98 [0.84; 1.15]	0	0	1.21 [0.85; 1.71]
unsupervised resistance	na	na	na	0	0	1.25 [0.77; 2.03]
vs. supervised aerobic						
supervised resistance	1	1	1.07 [0.87; 1.32]	3	0.51	0.95 [0.80; 1.14]
unsupervised aerobic	0	0	1.10 [0.92; 1.31]	2	1.00	1.30 [0.96; 1.75]
unsupervised resistance	na	na	na	0	0	1.35 [0.86; 2.11]
vs. supervised resistance						
unsupervised aerobic	0	0	1.03 [0.78; 1.35]	0	0	1.36 [0.96; 1.93]
unsupervised resistance	na	na	na	0	0	1.42 [0.88; 2.29]
vs. unsupervised aerobic						
unsupervised resistance	na	na	na	0	0	1.04 [0.75; 1.44]
vs. flexibility training						
no exercise	na	na	na	0	0	1.10 [0.84; 1.45]
supervised aerobic	na	na	na	1	0.38	1.02 [0.80; 1.31]
supervised resistance	na	na	na	1	0.4	0.98 [0.76; 1.25]

NOTE: NA: not available; prop: direct evidence proportion; NMA: network meta-analysis; LDL: low-density lipoprotein cholesterol

Appendix 6-25 subgroup analysis of diabetes duration in HDL

Comparisons	diabetes duration ≤6 (years)			diabetes duration ＞6 (years)		
	No. of direct comparisons	prop	NMA 95%-CI	No. of direct comparisons	prop	NMA 95%-CI
vs. Combined						
flexibility training	na	na	na	1	0.41	0.94 [0.85; 1.04]
no exercise	1	0.42	0.96 [0.82; 1.12]	2	0.84	1.03 [0.97; 1.09]
supervised aerobic	1	0.44	0.91 [0.78; 1.06]	1	0.25	0.97 [0.90; 1.05]
supervised resistance	1	0.44	0.98 [0.84; 1.14]	1	0.44	**0.85 [0.78; 0.93]**
unsupervised aerobic	0	0	0.98 [0.77; 1.23]	na	na	na
vs. no exercise						
supervised aerobic	2	0.62	0.95 [0.84; 1.07]	2	0.56	0.94 [0.87; 1.02]
supervised resistance	2	0.5	1.02 [0.90; 1.15]	0	0	**0.83 [0.75; 0.91]**
unsupervised aerobic	1	0.52	1.02 [0.83; 1.24]	na	na	na
vs. supervised aerobic						
supervised resistance	2	0.46	1.07 [0.94; 1.23]	2	0.61	**0.88 [0.81; 0.95]**
unsupervised aerobic	0	0	1.07 [0.86; 1.34]	na	na	na
vs. supervised resistance						
unsupervised aerobic	1	0.59	1.00 [0.82; 1.22]	na	na	na
vs. flexibility training						
no exercise	na	na	na	0	0	1.09 [0.98; 1.22]
supervised aerobic	na	na	na	1	0.38	1.03 [0.94; 1.14]
supervised resistance	na	na	na	1	0.52	0.90 [0.82; 0.99]

NOTE: NA: not available; prop: direct evidence proportion; NMA: network meta-analysis; HDL: high-density lipoprotein cholesterol

Appendix 6-25 subgroup analysis of study duration in HDL

Comparisons	Study duration ≤6 (month)			Study duration ＞6 (month)		
	No. of direct comparisons	prop	NMA 95%-CI	No. of direct comparisons	prop	NMA 95%-CI
vs. Combined						
flexibility training	1	0.42	0.94 [0.82; 1.07]	na	na	na
no exercise	2	0.6	1.00 [0.91; 1.11]	1	0.46	0.92 [0.84; 1.00]
supervised aerobic	1	0.25	0.92 [0.83; 1.02]	1	0.48	0.97 [0.89; 1.05]
supervised resistance	1	0.34	**0.89 [0.80; 0.99]**	1	0.51	0.96 [0.88; 1.04]
unsupervised aerobic	0	0	0.98 [0.84; 1.14]	0	0	0.72 [0.34; 1.50]
unsupervised resistance	0	0	0.90 [0.70; 1.17]	na	na	na
vs. no exercise						
supervised aerobic	3	0.67	0.91 [0.84; 1.00]	2	0.63	1.05 [0.97; 1.13]
supervised resistance	1	0.19	**0.89 [0.80; 0.98]**	1	0.45	1.04 [0.95; 1.13]
unsupervised aerobic	2	0.74	0.98 [0.86; 1.11]	0	0	0.78 [0.37; 1.62]
unsupervised resistance	0	0	0.90 [0.71; 1.15]	na	na	na
vs. supervised aerobic						
supervised resistance	3	0.53	0.97 [0.88; 1.07]	1	0.47	0.99 [0.91; 1.08]
unsupervised aerobic	0	0	1.07 [0.92; 1.23]	1	1	0.74 [0.36; 1.54]
unsupervised resistance	0	0	0.99 [0.76; 1.27]	na	na	na
vs. supervised resistance						
unsupervised aerobic	1	0.41	1.10 [0.96; 1.27]	0	0	0.75 [0.36; 1.56]
unsupervised resistance	0	0	1.02 [0.79; 1.31]	na	na	na
vs. unsupervised aerobic						
unsupervised resistance	1	1	0.92 [0.75; 1.14]	na	na	na
vs. flexibility training						
no exercise	0	0	1.07 [0.93; 1.23]	na	na	na
supervised aerobic	1	0.39	0.98 [0.86; 1.11]	na	na	na
supervised resistance	1	0.46	0.95 [0.83; 1.08]	na	na	na

NOTE: NA: not available; prop: direct evidence proportion; NMA: network meta-analysis; HDL: high-density lipoprotein cholesterol

Appendix 5- Subgroup analysis of type of patients in HDL

Comparisons	Sedentary				Non-sedentary			
	No. of direct comparisons	prop	nma	95%-CI	No. of direct comparisons	prop	nma	95%-CI
vs. Combined								
flexibility training	na	na	na		1	0.38	0.97	[0.86; 1.09]
no exercise	na	na	na		3	0.63	0.96	[0.89; 1.04]
supervised aerobic	na	na	na		2	0.4	0.97	[0.90; 1.05]
supervised resistance	na	na	na		2	0.43	0.92	[0.85; 1.00]
unsupervised aerobic	na	na	na		0	0	0.93	[0.79; 1.10]
unsupervised resistance	na	na	na		0	0	0.86	[0.67; 1.11]
vs. no exercise								
supervised aerobic	3	1	0.92	[0.78; 1.08]	2	0.43	1.01	[0.93; 1.10]
supervised resistance	0	0	0.92	[0.66; 1.28]	2	0.35	0.95	[0.88; 1.04]
unsupervised aerobic	1	1	1.02	[0.77; 1.35]	1	0.47	0.97	[0.83; 1.14]
unsupervised resistance	na	na	na		0	0	0.90	[0.70; 1.15]
vs. supervised aerobic								
supervised resistance	1	1	1.00	[0.75; 1.34]	3	0.48	0.95	[0.87; 1.03]
unsupervised aerobic	0	0	1.11	[0.81; 1.53]	1	0.05	0.96	[0.82; 1.13]
unsupervised resistance	na	na	na		0	0	0.89	[0.69; 1.14]
vs. supervised resistance								
unsupervised aerobic	0	0	1.11	[0.72; 1.72]	1	0.56	1.02	[0.87; 1.19]
unsupervised resistance	na	na	na		0	0	0.94	[0.73; 1.20]
vs. unsupervised aerobic								
unsupervised resistance	na	na	na		0	0	0.92	[0.76; 1.12]
vs. flexibility training								
no exercise	na	na	na		0	0	0.99	[0.88; 1.13]
supervised aerobic	na	na	na		1	0.37	1.00	[0.89; 1.13]
supervised resistance	na	na	na		1	0.44	0.95	[0.85; 1.06]

NOTE: NA: not available; prop: direct evidence proportion; NMA: network meta-analysis; HDL: high-density lipoprotein cholesterol

Appendix 6Absolute effect estimates of different exercise modalities using no exercise as baseline risk

Outcomes	Comparisons (vs. No exercise)	Number of studies and patients from direct evidence	RoM [95%CI]	Absolute effect estimates		Difference (95CI)	Ranking
HbA1c (%)	Combined	Direct evidence: 3 RCTs 443 Patients	0.93 (0.92,0.94)	6.98	7.51	0.53 Lower (0.60 Lower to 0.45 Lower)	0.99
	Supervised aerobic	Direct evidence:11 RCTs 802 Patients	0.96 (0.94,0.97)	7.21	7.51	0.30 lower (0.45 Lower to 0.23 Lower)	0.78
	Unsupervised aerobic	Direct evidence: 2 RCTs, 135 Patients	1.03 (1.00,1.07)	7.74	7.51	0.23 Higher (0 Lower to 0.53 Higher)	0.18
	Supervised resistance	Direct evidence: 5 RCTs, 413 Patients	0.96 (0.95,0.98)	7.21	7.51	0.30 Lower (0.38 Lower to 0.15 Lower)	0.70
	Unsupervised resistance	Direct evidence: 2 RCTs, 48 Patients	1.03 (0.99,1.07)	7.74	7.51	0.23 Higher (0.08 Lower to 0.53 Higher)	0.23
	Flexibility training	No direct evidence, indirect evidence only	0.92 (0.86,0.98)	6.91	7.51	0.60 Lower (1.05 Lower to 0.15 Lower)	0.18
FBG (mg/dl)	Combined	Direct evidence: 1 RCTs 36 Patients	0.93 (0.82, 1.07)	124	134	9.38 Lower (24.12 Lower to 9.38 Higher)	0.71
	Supervised aerobic	Direct evidence: 5 RCTs 253 Patients	0.93 (0.88,0.98)	124	134	9,38 Lower (16.08 Lower to 2.68 Lower)	0.82
	Unsupervised aerobic	No direct evidence, indirect evidence only	1.11 (0.81,1.52)	148.74	134	14.74 Higher (25.46 Lower to 69.68 Higher)	0.19
	Supervised resistance	Direct evidence: 2 RCTs, 50 Patients	1.01 (0.92,1.12)	135.34	134	1.34 Higher (10.72 Lower to 16.08 Higher)	0.32
	Unsupervised resistance	No direct evidence, indirect evidence only	1.03 (0.72,1.47)	138.02	134	4.02 Higher (37.52 Lower to 62.98 Higher)	0.44
	Anaerobic	No direct evidence, indirect evidence only	0.95 (0.88,1.03)	127.30	134	6.70 Lower (16.08 Lower to 4.02 Higher)	0.64

Weight (kg)	Combined	Direct evidence: 2 RCTs 77 Patients	0.94 (0.89,0.99)	78.68	83.70	5.02 Lower (9.21 Lower to 0.84 Lower)	0.86
	Supervised aerobic	Direct evidence:6 RCTs 211 Patients	0.98 (0.95,1.01)	82.03	83.70	1.67 Lower (4.19 Lower to 0.84 Higher)	0.48
	Unsupervised aerobic	Direct evidence: 1 RCTs, 75 Patients	1.04 (1.00,1.09)	87.05	83.70	3.35 Higher (0 Lower to 7.53 Higher)	0.01
	Supervised resistance	Direct evidence: 3 RCTs, 90 Patients	0.98 (0.95,1.02)	82.03	83.70	1.67 Lower (4.19 Lower to 1.67 Higher)	0.47
	Unsupervised resistance	Direct evidence: 1 RCTs, 28 Patients	0.97 (0.91,1.03)	81.19	83.70	2.51 Lower (7.53 Lower to 2.51 Lower)	0.61
	Anaerobic	No direct evidence, indirect evidence only	0.94 (0.86,1.02)	78.68	83.70	5.02 Lower (11.72 Lower to 1.67 Lower)	0.82
SBP (mmHg)	Combined	Direct evidence: 2 RCTs 186 Patients	0.97 (0.93,1.01)	126.10	130	3.90 Lower (9.10 Lower to 1.30 Higher)	0.59
	Supervised aerobic	Direct evidence: 6 RCTs 365 Patients	0.97 (0.95,1.00)	126.10	130	3.90 Lower (6.50 Lower to 0 Higher)	0.48
	Unsupervised aerobic	Direct evidence: 2 RCTs, 95 Patients	0.97 (0.93,1.01)	126.10	130	3.90 Lower (9.10 Lower to 1.30 Higher)	0.58
	Supervised resistance	Direct evidence: 3 RCTs, 60 Patients	0.96 (0.93,0.99)	124.80	130	5.20 Lower (9.10 Lower to 1.30 Lower)	0.69
	Unsupervised resistance	No direct evidence, indirect evidence only	0.95 (0.87,1.04)	123.50	130	6.50 Lower (16.90 Lower to 5.20 Higher)	0.69
	Anaerobic	No direct evidence, indirect evidence only	0.99 (0.90,1.08)	128.70	130	1.30 Lower (13.00 Lower to 10.40 Higher)	0.37
DBP (mmHg)	Combined	Direct evidence: 2	0.99	79.20	80		0.4

		RCTs 165 Patients	(0.94,1.04)			0.80 Lower (4.80 Lower to 3.20 Higher)	
	Supervised aerobic	Direct evidence: 6 RCTs 370 Patients	0.97 (0.94,1.00)	77.60	80	2.40 Lower (4.80 Lower to 0 Higher)	0.65
	Unsupervised aerobic	Direct evidence: 2 RCTs, 95 Patients	0.94 (0.89,1.00)	75.20	80	4.80 Lower (8.80 Lower to 0 Higher)	0.85
	Supervised resistance	Direct evidence: 3 RCTs, 164 Patients	0.97 (0.93,1.00)	77.60	80	2.40 Lower (5.60 Lower to 0 Lower)	0.67
	Unsupervised resistance	No direct evidence, indirect evidence only	0.97 (0.86,1.09)	77.60	80	2.40 Lower (11.20 Lower to 7.20 Higher)	0.57
	Anaerobic	No direct evidence, indirect evidence only	1.04 (0.96,1.13)	83.20	80	3.20 Higher (13.00 Lower to 10.40 Higher)	0.1
TC (mg/dl)	Combined	Direct evidence: 2 RCTs 74 Patients	1.03 (0.97,1.08)	189.52	184	5.52 Higher (5.52 Lower to 14.72 Higher)	0.06
	Supervised aerobic	Direct evidence: 4 RCTs 203 Patients	0.89 (0.85,0.94)	163,76	184	20.24 Lower (27.60 Lower to 11.04 Lower)	0.84
	Unsupervised aerobic	Direct evidence: 2 RCTs, 95 Patients	0.96 (0.90,1.02)	176.64	184	7.36 Lower (18.40 Lower to 3.68 Higher)	0.47
	Supervised resistance	Direct evidence: 1 RCTs, 20 Patients	0.88 (0.83,0.94)	161.92	184	22.08 Lower (31.28 Lower to 11.04 Lower)	0.92
	Flexibility training	No direct evidence, indirect evidence only	0.95 (0.86,1.05)	174.80	184	9.20 Lower (25.76 Lower to 9.20 Higher)	0.51
TG (mg/dl)	Combined	Direct evidence: 4 RCTs 328 Patients	0.75 (0.67,0.84)	111.60	184	37.20 Lower (49.10 Lower to 23.81 Lower)	1
	Supervised	Direct evidence: 6	0.87	129.46	184		0.63

	aerobic	RCTs 453 Patients	(0.80,0.96)	19.34 Lower (29.76 Lower to 5.95 Lower)		
	Unsupervised aerobic	Direct evidence: 2 RCTs, 95 Patients	0.95 (0.86.1.05)	141.36	184	0.25
				7.44 Lower (20.83 Lower to 7.44 Higher)		
	Supervised resistance	Direct evidence: 3 RCTs, 270 Patients	0.89 (0.81,0.97)	132.43	184	0.58
				16.37 Lower (28.27 Lower to 4.46 Lower)		
LDL (mg/dl)	Combined	Direct evidence: 3 RCTs 201 Patients	0.92 (0.81,1.05)	99.36	108	0.59
				8.64 Lower (20.52 Lower to 5.40 Higher)		
	Supervised aerobic	Direct evidence: 5 RCTs 330 Patients	0.89 (0.80,0.99)	96.12	108	0.75
				11.88 Lower (21.60 Lower to 1.08Lower)		
	Unsupervised aerobic	Direct evidence: 1 RCTs, 75 Patients	1.08 (0.88,1.33)	116.64	108	0.18
				8.64 Higher (12.96 Lower to 35.64 Higher)		
	Supervised resistance	Direct evidence: 1 RCTs, 123 Patients	0.88 (0.76,1.01)	95.04	108	0.79
				12.96 Lower (25.92 Lower to 1.08 Higher)		
	Unupervised resistance	No direct evidence, indirect evidence only	1.12 (0.81,1.56)	120.96	108	0.17
				12.96 Higher(20.52 Lower to 60.48 Higher)		
	Flexibility training	No direct evidence, indirect evidence only	0.89 (0.71,1.10)	96.12	108	0.71
				11.88 Lower (21.32 Lower to 10.80 Higher)		
HDL (mg/dl)	Combined	Direct evidence: 3 RCTs 201 Patients	0.97 (0.94,1.00)	44.43	45.80	0.19
				1.37 Lower (2.75 Lower to 0.00 Lower)		
	Supervised aerobic	Direct evidence: 5 RCTs 330 Patients	0.92 (0.89,0.96)	40.76	43.97	0.60
				3.66 Lower (5.04 Lower to 1.83 Lower)		
	Unsupervised aerobic	Direct evidence: 2 RCTs, 95 Patients	0.99 (0.87,1.12)	45.34	45.80	0.44
				0.46 Higher (5.95 Lower to 5.50 Higher)		
	Supervised	Direct evidence: 2	0.90	38.93	43.05	0.74

	resistance	RCTs, 143 Patients	(0.85,0.94)	4.58 Lower (6.87 Lower to 2.75 Lower)		
	Unsupervised resistance	No direct evidence, indirect evidence only	0.91 (0.71,1.17)	41.68	45.80	0.72
				4.12 Higher(13.28 Lower to 7.79 Higher)		
	Flexibility training	No direct evidence, indirect evidence only	0.98 (0.86,1.13)	44.88	45.80	0.47
				0.92 Lower (6.41 Lower to 5.95 Higher)		
